# Elevated signal transducers and activators of transcription 1 correlates with increased C-C motif chemokine ligand 2 and C-X-C motif chemokine 10 levels in peripheral blood of patients with systemic lupus erythematosus

**DOI:** 10.1186/ar4448

**Published:** 2014-01-23

**Authors:** Paul R Dominguez-Gutierrez, Angela Ceribelli, Minoru Satoh, Eric S Sobel, Westley H Reeves, Edward KL Chan

**Affiliations:** 1 Department of Oral Biology, University of Florida, P.O. Box 100424, 1395 Center Drive, Gainesville, FL 32610-0424, USA; 2 Division of Rheumatology and Clinical Immunology, Department of Medicine, University of Florida, P.O. Box 100221, 1600 SW Archer Rd, Gainesville, FL 32610-0221, USA; 3 School of Health Sciences, University of Occupational and Environmental Health, Japan, 1-1 Isei-ga-oka, Yahata-nishi-ku, Kitakyushu, Fukuoka 807-8555, Japan; 4 Current address: Rheumatology and Clinical Immunology, Humanitas Clinical and Research Center, Via A. Manzoni 56, 20089 Rozzano, Italy; 5 Current address: BIOMETRA Department, University of Milan, Milan, Italy; 6 Current address: Department of Urology, University of Florida, 1600 SW Archer Road, Gainesville, FL 32610-0247, USA

## Abstract

**Introduction:**

The present study examines the levels of recently reported biomarkers, adenosine deaminase acting on RNA (ADAR), C-C motif chemokine ligand 2 (CCL2), C-X-C motif chemokine 10 (CXCL10), signal transducers and activators of transcription 1 (STAT1), and miR-146a in systemic lupus erythematosus (SLE) patients over multiple visits.

**Methods:**

Peripheral blood leukocytes were collected from 65 healthy donors and 103 SLE patients, 60 of whom had samples from 2 or more visits. Total RNA was isolated and analyzed for the expression of mRNA and microRNA using Taqman real time PCR assays. Relative expression of I-IFN signature genes, chemokines, and miR-146a were determined by the ^ΔΔ^CT method. Results were correlated with clinical data and analyzed by Wilcoxon/Kruskal-Wallis test and Fisher’s exact test.

**Results:**

Levels of ADAR, CCL2, CXCL10, and STAT1 in SLE were significantly elevated compared with the healthy controls (*P* <0.0001). ADAR, CCL2, and CXCL10 showed significant correlation with IFN score in both healthy donors (*P* <0.0033) and SLE patients (*P* <0.0001). In SLE patients, miR-146a level was not significantly different from healthy controls nor correlated to the IFN score. Two STAT1 populations were identified: a low STAT1 and a high STAT1 group. High STAT1 patient visits displayed higher (*P* ≤0.0020) levels of CCL2 and CXCL10 than the low STAT1 patient visits. STAT1 levels correlated with IFN score in low STAT1 group but not in high STAT1 group. More importantly, high STAT1 levels appeared as an enhancer of CCL2 and CXCL10 as indicated by the significantly stronger correlation of CCL2 and CXCL10 with IFN score in high STAT1 patient visits relative to low STAT1 patient visits.

**Conclusion:**

Our data indicate a novel role for STAT1 in the pathogenesis of SLE as an expression enhancer of CCL2 and CXCL10 in SLE patients with high levels of STAT1. Future study is needed to examine the exact role of STAT1 in the etiology of SLE.

## Introduction

Systemic lupus erythematosus (SLE) is a chronic systemic autoimmune disease characterized by periods of increased disease activity, referred to as flare-ups, and periods of remission. Several genetic and environmental factors have been implicated in SLE etiopathogenesis, but in recent years increased type I interferon (IFN-I, IFNα and IFNβ) expression has been discovered to play a key role in the majority of SLE patients, despite being known for over 30 years that it is elevated in SLE patients [1-4]. Because of the technical challenges in measuring the numerous isoforms of IFNα, one common way to evaluate IFN-I expression is to examine the levels of common IFN-inducible genes, such as 2′,5′-oligoadenylate synthetase (OAS1), myxovirus resistance 1 (MX1), and lymphocyte antigen 6 complex locus E (LY6E); the mRNA levels of these IFN-I-inducible genes are then used to calculate the IFN score [1,5-7]. Another interferon inducible gene that plays an important antiviral and immunomodulatory function is the adenosine deaminase acting on RNA (ADAR). ADAR is an enzyme that catalyzes the conversion from adenosine (A) to inosine (I) in double-stranded RNA (dsRNA) substrate [8,9], with an impact on RNA at different levels, such as mRNA splicing and degradation [10,11]. Furthermore, ADAR1 has been observed to suppress interferon regulatory factor (IRF)3 and protein kinase RNA-activated (PKR) and therefore blocking IFN induction [12-14]. The ability of ADAR1 to respond and regulate IFN-I production makes it an intriguing IFN-I-inducible gene to examine in SLE. Up to now, ADAR1 expression has only been observed in T-cells of SLE patients, as shown in a limited number of studies [15-17]. In fact, Laxminarayana *et al*. showed that ADAR1 is upregulated approximately 3-fold in SLE patients [15]. The same group later observed the increased editing of ADAR2 by ADAR1 in T-cells of SLE patients [16]. Additionally, due to increased ADAR1 in SLE patients, Orlowski *et al*. observed an increase of phosphodiesterase 8A1, which participates in the termination of cyclic nucleotide signaling by hydrolyzing cAMP and cGMP and is activated by IFN and enhances T-cell adhesion [17].

Other IFN-I-inducible genes include signal transducers and activators of transcription (STAT)1 and 2. STAT1 is involved in type I, II, and III IFN signaling and has been observed to be elevated in SLE [18]. In response to type I IFN, STAT1 causes IFN receptor (IFNAR)1 and 2 dimerization, activation and phosphorylation of IFNAR by Tyk2 and Jak1, and thus docking and phosphorylation of STAT1 and STAT2 [19]. The heterodimer STAT1-STAT2 is then translocated into the nucleus where it can bind specific promoters playing a key role in IFN signaling and production [20].

Besides STAT1 and ADAR, IFN-regulated chemokines have become another important topic of research in recent years [21]. Two of these chemokines have been shown to be SLE biomarkers, and they are called C-C motif chemokine ligand 2 (CCL2) and C-X-C motif chemokine 10 (CXCL10) [22]. CCL2, formerly referred to as monocyte chemotactic protein-1 (MCP-1), is a potent recruiter of monocytes, T-cells, basophils, and dendritic cells to the site of infection or tissue damage, but it has no effect on neutrophils or eosinophils unless the N-terminus of CCL2 is cleaved [18,23]. Some cell types such as monocytes, macrophages, and dendritic cells can primarily secrete CCL2, which signals via the cell surface receptors CCR2 and CCR4 and is upregulated by IFNα and IFNβ [24,25]. The role of CCL2 is beneficial in clearing pathogens, but it has also been involved in some pathological processes. In a glomerulonephritis mouse model, CCL2 plays a role in crescent formation and interstitial fibrosis supported by the observation that anti-CCL2 antibodies can reduce crescent formation, renal impairment, and scarring, as well as T cell and macrophage infiltration [26]. CCL2 has been observed in the recruitment of T cells and monocytes/macrophages in lupus nephritis and blockade of CCL2 ameliorates lupus nephritis in MRL-(Fas)lpr mice [23,27]. In a serologic proteome study by antibody microarray in SLE, CCL2 was identified as one of the twelve upregulated proteins; furthermore CCL2 was one of three chemokines that would precede lupus flare, indicating that they are good predictors of increased SLE activity [21].

CXCL10, also known as IFN gamma-induced protein 10 (IP-10), is a chemokine of the C-X-C motif family. Similar to CCL2, CXCL10 is a potent attractor of monocytes, macrophages, T-cells, natural killer (NK) cells, and dendritic cells to sites of tissue damage and infection [28,29]. CXCL10 is an IFN-response cytokine that binds its CCL3 receptor and acts via Jak/STAT pathway activation [30-32]. Even though CXCL10 is a potent immune responder for bacterial and viral infections and a critical biomarker for organ transplant rejection, its role in the pathogenesis of autoimmune diseases is not clear [33,34]. Furthermore, the combination of CXCL10 and CCL2 protein levels could be useful as prediction factor for upcoming flares [22].

The reason behind upregulation and control of IFN in SLE is not known, but some studies have recently focused on the possible role played by selected microRNAs (miRNAs). MiRNAs are small non-encoding 20- to 23-nucleotide-long RNAs, that regulate their target mRNA by binding to the 3′ UTR, causing translational repression and/or degradation of targets. miR-146a is one of the most significant miRNAs in regulating innate immune response and tolerance [35] and it was first shown to be involved in toll-like receptor (TLR) regulation through the nuclear factor (NF)-кB pathway [36]. miR-146a would function to attenuate the immune response and regulate inflammation in normal immune response and autoimmune disorders, and it is also a critical regulator of endotoxin-induced tolerance and cross-tolerance [37-39]. To date, miR-146a has been found in association with autoimmune diseases such as Sjögren’s syndrome [40], psoriasis [41,42], and rheumatoid arthritis [43-45].

Tang *et al*. reported that miR-146a was under-expressed in peripheral blood mononuclear cells (PBMCs) of Chinese SLE patients [46]. miR-146a was significantly lower in patients with active SLE with proteinuria compared to those with inactive SLE [46]. Additionally, SLE patients displayed an inverse correlation between miR-146a expression and IFN score [46]. Tang *et al.* also demonstrated that reduction of miR-146a may enhance the signaling due to elevated levels of STAT1 and IRF5 which leads to increased production of IFN [46]. The reduced levels of miR-146a observed in Chinese SLE patients could potentially explain elevation of IFN by loss of regulation of STAT1 expression.

Our present study evaluates the interaction among STAT1, ADAR, CCL2, CXCL10, and miR-146a in SLE patients and healthy controls, demonstrating that all except for miR-146a correlate with IFN score in both SLE patients and healthy donors.

## Methods

### Healthy donors’ and SLE patients’ demographic data

Whole blood was collected from a total of 103 SLE patients and 65 healthy controls enrolled in the University of Florida Center for Autoimmune Diseases registry from 2008 to 2011. Healthy donors (HD) were selected based on no history of autoimmune disease, and all SLE patients satisfied the American College of Rheumatology (ACR) criteria [47]. Healthy donors only visited the clinic once, therefore, they represent a single visit. There were a total of 180 SLE visits with sequential samples collected in 60 SLE patients (Table 1). SLE patients and healthy controls were segregated by ethnic profile (Table 1). All human blood samples were obtained from enrolled individuals with the approval of the institutional review board (IRB) at the University of Florida. This study meets and is in compliance with all ethical standards in medicine and informed consent was obtained from all patients according to the Declaration of Helsinki.

**Table 1:** Demographic data of SLE patients and healthy donors

	**SLE**	**HD**
Number of cases	103	65
Number of visits (2 or more)	180 (60)	65 (0)
Mean age, years (range)	44 (25–68)	33 (19–59)
Sex, female/male/unknown, n	90/13/0	31/9/24
Race, AA/EA/LA/AsA/IrA/unknown^1^, n	35/50/12/3/3/0	10/18/6/3/2/25
Race by visit (AA/EA/LA/AsA/IrA/unknown)^1^, n	64/86/20/7/3/0	10/18/6/3/2/25
Active/inactive by SLEDAI, number of patients	49/131	N/A
**Main clinical features, number of patients**		
Malar rash	9/94	N/A
Discoid lesions	2/101	N/A
Photosensitivity	8/95	N/A
Oral ulcers	7/96	N/A
Arthritis	17/86	N/A
Serositis	4/99	N/A
Pleuritis	5/98	N/A
Nephritis	49/54	N/A
Seizures	2/101	N/A
Psychosis	1/102	N/A

^1^AA, African Americans; AsA, Asian Americans; EA, European Americans; HD, healthy donors with no history of autoimmune disease; IrA, interracial Americans; LA, Latino Americans; unknown, undisclosed race; N/A, not applicable.

### Leukocytes and RNA purification

Peripheral blood leukocytes were collected from whole blood using Ambion LeukoLOCK kit (Ambion, Austin, TX, USA). LeukoLOCK filters were washed twice with 3 ml of PBS and stabilized with 3 ml of RNAlater solution. Stabilized filters were stored for a minimum of 24 h at −80°C before collecting total RNA. Total RNA, including small RNAs, was collected using the “Alternative Protocol” (version 0602, Ambion) for the extraction of RNA from cells captured on LeukoLOCK filters using TRI reagent.

### mRNA and microRNA quantitative RT-PCR

OAS1 (Hs00973637_m1), MX1 (Hs00895608_m1), LY6E (Hs00158942_m1), STAT1 (Hs01013996_m1), CCL2 (Hs00234140_m1), CXCL10 (Hs00171042_m1), ADAR (Hs00241666_m1), TNFα (Hs00174128_m1), and pri-miR-146a (Hs033303259_pri) levels were analyzed by TaqMan mRNA assay primers (Applied Biosystems, Foster City, CA, USA). mRNA qRT-PCR was performed as a duplex with 18S rRNA assayed as the normalizer. mRNA was transcribed to cDNA using the TaqMan High-Capacity cDNA Reverse Transcription Kit followed by quantitative (q)PCR using TaqMan Fast Advance PCR Master Mix (Applied Biosystems). miR-146a (000468; Catalogue # 4427975) was analyzed by miRNA qRT-PCR using the TaqMan MicroRNA Reverse Transcription Kit, TaqMan Fast Advance PCR Master Mix, and TaqMan MicroRNA primers (Applied Biosystems). All reactions were analyzed using StepOne Real-Time PCR System (Applied Biosystems).

### Anti-dsDNA ELISA

After the collection of leukocytes with the LeukoLOCK filters, the leukocyte free blood was transferred to 10 ml Vacutainer SST plus blood-collection tubes (BD, Franklin Lakes, NJ, USA). Blood was centrifuged at 1,000 g for 20 minutes. The plasma was transferred to a 15-ml conical tube and stored at −20°C. Anti-dsDNA ELISA was performed as previously described [48]. In brief, anti-human IgG secondary antibody was used and samples were considered positive when the absorbance was greater than the mean plus three SD from the healthy controls.

### Complement levels

C3 and C4 complement levels were obtained from clinical data. C3 levels lower than 90 mg/dl and C4 levels less than 15 mg/dl were considered as low complement levels in the analysis.

### IFN score and SLE activity

The expression of three known type-I IFN signature genes, MX1, OAS1, and LY6E, was z-transformed into IFN score as previously shown [1,49]. The SLE disease activity index (SLEDAI) was used to classify the patients into active (SLEDAI >4) or inactive (SLEDAI ≤4) at the time of the visit (Table 1) [50,51].

### Cell culture and innate immune ligand stimulation

Human THP-1 cells were obtained from the American Type Culture Collection (ATCC, Manassas, VA, USA). THP-1 cells were maintained in RPMI containing 10% (v/v) FBS (Mediatech, Manassas, VA, USA) and 100 U/ml penicillin-streptomycin (Mediatech). For analysis of THP-1 monocyte response to ligand *in vitro*, log-phase cells were seeded at 5 × 10^5^ cells/ml in a 24-well plate. Cells were stimulated with the following agonists: 1,000 ng/ml of lipopolysaccharide from *S. enterica* serotype Minnesota Re595 (LPS Se, TLR4 ligand, Ultrapure grade, Sigma-Aldrich Corp. St. Louis, MO USA), 0.10 and 1.0 ng/ml IFNα2 (PBL Interferon Source, Piscataway, NJ, USA), and 0.10 or 1.0 ng/ml IFNβ (PBL Interferon Source). TLR4 ligands were reconstituted in endotoxin-free water and used at concentrations as reported before [38]. IFNα2 and IFNβ were reconstituted in endotoxin-free PBS with 1 mg/ml BSA to make 5-μg/ml stocks stored at −80°C.

### Data analysis

The copy number of miR-146a was normalized to total loaded RNA, whereas mRNA levels were normalized to 18S RNA. The copy number of miR-146a was determined using a standard curve with synthetic miR-146a (Integrated DNA Technologies Inc., Coralville, IA, USA) [52]. Relative expression of mRNA compared to controls was determined by the ^ΔΔ^C_T_ (cycle threshold) method [53]. Analyses were performed using SAS version 9.2 and JMP Genomics version 5 (SAS, Cary, NC, USA). The Wilcoxon/Kruskal-Wallis test was used to evaluate significance between groups, whereas the Wilcoxon signed rank test for matched pairs was used to evaluate SLE patients with two visits. *P*-values <0.05 were considered significant. Before applying ordinary linear regression analyses, the distributions of datasets were confirmed for normality. The coefficient of determination (*r*^2^) was used to determine linear correlation. Significant differences between slopes was evaluated by analysis of covariance (ANCOVA). The Generalized Estimating Equation (GEE) model for Repeated Measures was used to account for possible with-in subject effects from patients with multiple visits [54].

## Results

### Expression of candidate biomarkers in the SLE cohort

**Figure 1:**
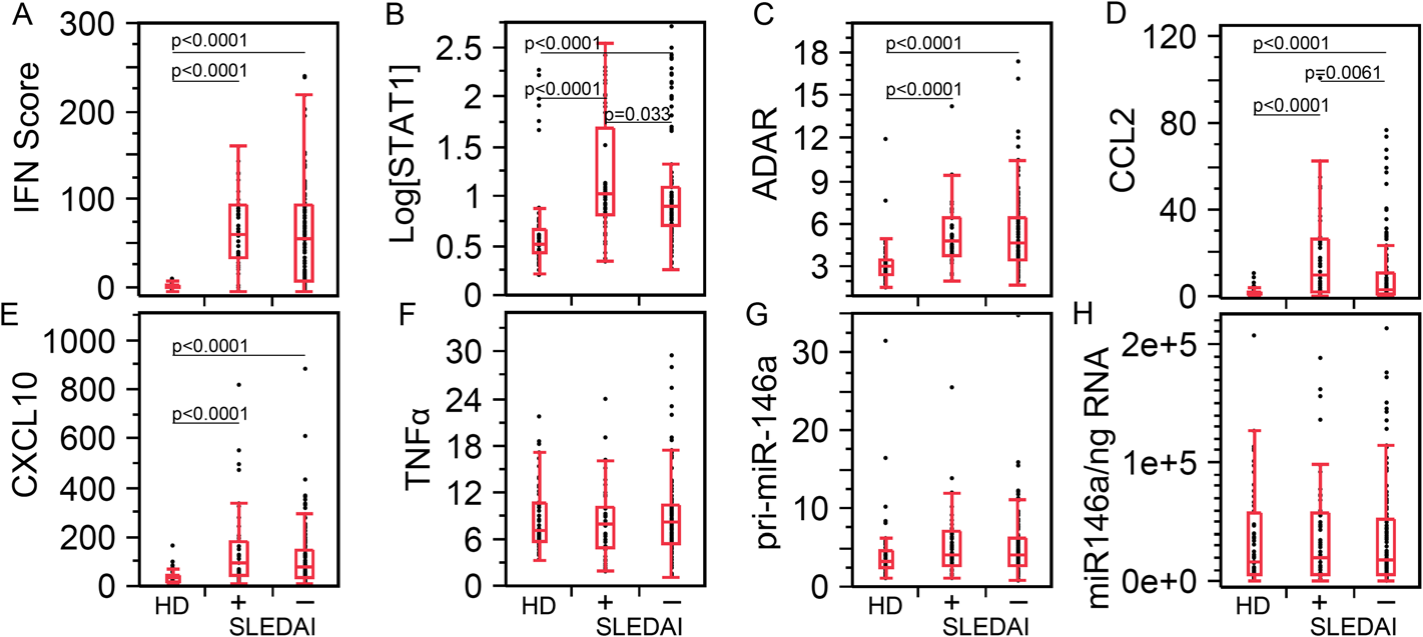
**Correlation of IFN score, STAT1, ADAR, CCL2, CXCL10, and miR-146a levels to systemic lupus erythematosus (SLE) disease activity. (A)** IFN score, **(B)** STAT1, **(C)** ADAR, **(D)** CCL2, and **(E)** CXCL10 were significantly elevated in SLE patient visits (active and inactive disease activity are indicated as + and - respectively) compared to healthy donors (HD). No statistical difference was detected between active and inactive SLE for IFN score, ADAR, or CXCL10. **(F-H)** TNFα, pri-miR-146a, and miR-146a did not show any significant difference among the groups. STAT, signal transducers and activators of transcription; ADAR, adenosine deaminase acting on RNA; CCL2, C-C motif chemokine ligand 2; CXCL10, C-X-C motif chemokine 10.

To determine whether previously reported biomarkers were elevated in our SLE patient cohort, we measured the biomarker expression levels in HD, active SLE, and inactive SLE patient visits (Figure 1). The SLE cohort was segregated by SLEDAI into active SLE (49 visits, SLEDAI >4) and inactive SLE (131 visits, SLEDAI ≤4). The level of IFN-I was estimated by quantifying the expression of IFN-inducible genes. The IFN score, STAT1, ADAR, CCL2, and CXCL10 levels were significantly elevated at both active and inactive SLE patient visits compared to HD (Figure 1A-E), establishing and confirming that these biomarkers were aberrantly overexpressed in our SLE patients. To explore if these biomarkers were capable of distinguishing disease activity status, active and inactive patient visits were compared to one another. No significant difference was observed between active and inactive SLE patient visits for IFN score (Figure 1A, mean ± SD, 62.7 ± 6.1 units versus 57.8 ± 4.9 units), ADAR (Figure 1C, 5.27 ± 0.31 fold versus 5.27 ± 0.23 fold), and CXCL10 (Figure 1E, 158.1-fold ± 26.6 versus 120.0-fold ± 10.5), but STAT1 (Figure 1B, 44.8 ± 10.7 vs 34.4 ± 6.6 fold, *P* = 0.033) and CCL2 (Figure 1D, 18.2-fold ± 3.1 versus 9.96-fold ± 1.42, *P* = 0.0061) were significantly elevated in active SLE compared to inactive SLE patient visits. TNFα, which is not generally involved in the pathogenesis of SLE, was used as a negative control. As expected, TNFα was not significantly different among the three groups (Figure 1F). Similarly miR-146a did not display any significant difference among active SLE, inactive SLE, and HD (Figure 1G). To validate this, we determined the levels of the primary transcript of miR-146a (pri-mir-146a) which also did not demonstrate any significant difference among active SLE, inactive SLE, and HD. With the exception of miR-146a, these results are consistent with reports on SLE patients with elevated IFN score compared to HD [1,49] as well as upregulated levels of IFN signature genes (STAT1 and ADAR) [15-17] and chemokines (CCL2 and CXCL10) [21].

**Figure 2:**
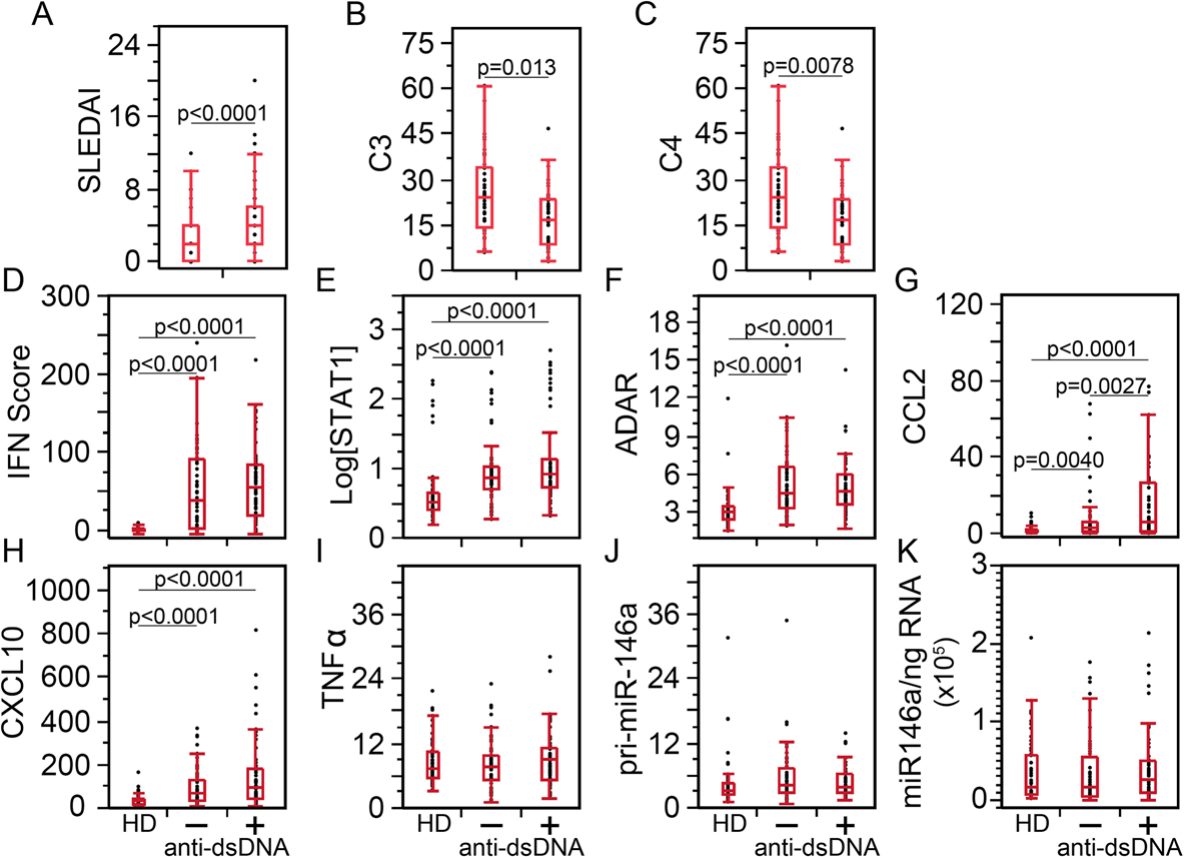
**Correlation of IFN score, STAT1, ADAR, CCL2, CXCL10, and miR-146a levels to anti-double-strandard DNA (dsDNA) autoantibodies. (A)** Systemic lupus erythematosus disease activity (SLEDAI) scores were significantly higher in anti-dsDNA(+) than anti-dsDNA(−) patient visits. **(B**, **C)** C3 and C4 were significantly lower in anti-dsDNA(+) than in anti-dsDNA(−) patients. **(D)** IFN score, **(E)** STAT1, **(F)** ADAR, **(G)** CCL2, and **(H)** CXCL10 were significantly elevated in SLE patient visits compared to healthy donors (HD), but no statistical differences were detected between anti-dsDNA(+) and anti-dsDNA(−) patients for IFN score, ADAR, or CXCL10. **(I**-**K)** TNFα, pri-miR-146a, and miR-146a did not show any significant difference among the groups. STAT, signal transducers and activators of transcription; ADAR, adenosine deaminase acting on RNA; CCL2, C-C motif chemokine ligand 2; CXCL10, C-X-C motif chemokine 10.

The clinical and expression data were correlated with anti-dsDNA autoantibody level, which is an indicator for patients’ disease activity in certain patients [55-58]. Decreases in C3 and C4 levels correlated with SLE activity and renal damage as well as increased levels of anti-dsDNA antibodies [59]. Anti-dsDNA autoantibody levels have also been used for sub-classification of SLE patients [60,61]. SLE patient visits and HD were segregated into anti-dsDNA(+) and anti-dsDNA(−). Patient visits that were anti-dsDNA(+) displayed higher SLEDAI and decreased C3 and C4 levels (Figure 2A-C). The results for the remaining biomarkers (Figure 2D-K) closely resembled those from active versus inactive SLEDAI results (Figure 1).

The influence of race in anti-dsDNA, IFN score, STAT1, CCL2, and CXCL10 were also examined. African Americans (AA) and European Americans (EA) contributed to 83.3% of the visits, followed by Latin Americans (LA) and Asian Americans (AsA) for 15%, and interracial Americans (IrA) for less than 2% of patient visits (Table 1). Due to the small sample size, IrA were excluded in all subsequent analyses. In general, results show that higher levels of anti-dsDNA, IFN score, STAT1, CCL2, and CXCL10 were observed in all race groups analyzed (Additional file 1: Figure S1). The lack of statistically significant differences between SLE and HD in certain groups, such as LA, might be due to the smaller sample sizes.

By comparing patients of different race (Additional file 1: Figure S2), the levels of the parameters examined were all higher in AA than EA, LA, and AsA. In particular, AA had significantly higher SLEDAI (*P* = 0.024), anti-dsDNA level (*P* = 0.044), IFN score (*P* = 0.0005), STAT1 (*P* = 0.0011), CCL2 (*P* = 0.0004), and CXCL10 (*P* = 0.0004) than EA. Furthermore, AA had significantly (*P* ≤0.014) higher IFN score, STAT1, CCL2, and CXCL10 than LA (Additional file 1: Figure S2B-F). Also in this case, the lack of additional statistically significant results for LA and AsA might be due to the small sample sizes. However, AA clearly displayed increased biomarker levels more than any other race.

### Biomarker interrelationship in SLE patients with return visits

**Figure 3:**
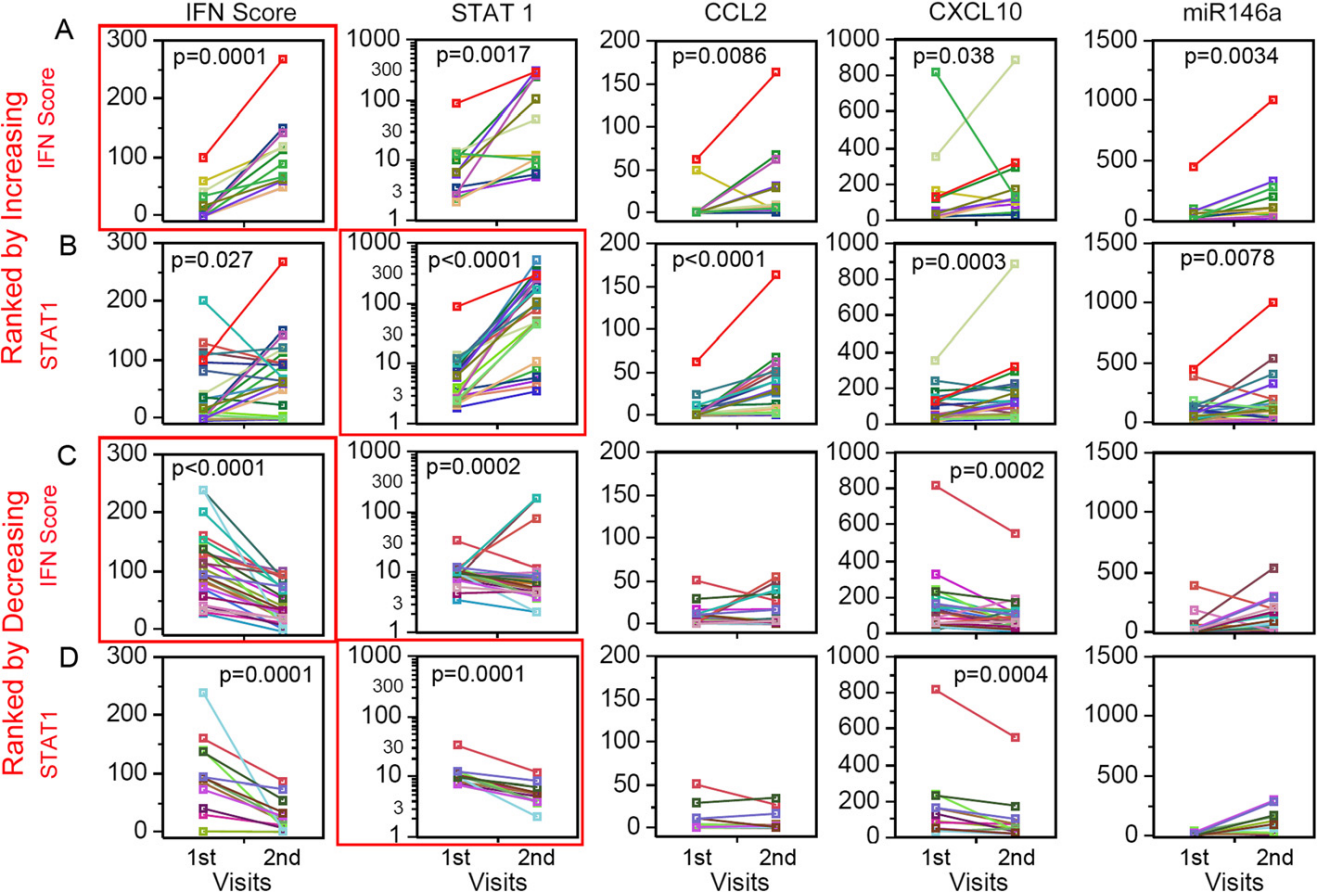
**Systemic lupus erythematosus (SLE) patients with two visits ranked by increasing or decreasing IFN score and STAT1.** Data from the first and second visits for each patient is denoted by an individual color line. **(A)** SLE patients ranked by increasing IFN score from the first to the second visit showed significant increase in STAT1, CCL2, CXCL10, and miR-146a. **(B)** Patients ranked by increasing STAT1 also showed significant increase in IFN score, CCL2, CXCL10, and miR-146a. **(C)** SLE patients ranked by decreasing IFN score from the first to the second visit showed significant decrease only in STAT1 and CXCL10. **(D)** Patients ranked by decreasing STAT1 showed significant decrease in IFN score and CXCL10. STAT, signal transducers and activators of transcription; CCL2, C-C motif chemokine ligand 2; CXCL10, C-X-C motif chemokine 10.

To expand upon the interrelationship of these biomarkers, data from SLE patients with two consecutive visits were segregated for analyses by increasing or decreasing IFN score by at least 50% from the first to the second visit. Patients with increasing IFN score from one visit to the next (n =13; *P* = 0.0001, Figure 3A) displayed a significant increase in STAT1 (*P* = 0.0017), CCL2 (*P* = 0.0086), CXCL10 (*P* = 0.038), and miR-146a (*P* = 0.0034). Similarly, for SLE patients with increasing STAT1 by at least 50% between the first and second visit (n = 25; *P* <0.0001, Figure 3B), significant increases were observed for IFN score (*P* = 0.027), CCL2 (*P* <0.0001), CXCL10 (*P* = 0.0003), and miR-146a (*P* = 0.0078). The strong correlation between STAT1, CCL2, and CXCL10 were expected; however, correlation between IFN score and increasing STAT1 was weaker than expected. This may be indicating that high STAT1 levels do not necessarily translate into high levels of IFN-I. The highly significant correlation between miR-146a levels and IFN score in the return visits was unexpected, as the level of miR-146a in SLE was not significantly different from HD (Figures 1H and 2H) and also it was previously reported to be decreased in SLE and inversely correlated with IFN score in a Chinese SLE cohort [46].

SLE patients who had decreasing IFN score by at least 50% between first and second visit (N = 32; *P* <0.0001, Figure 3C) displayed a significant decrease in STAT1 (*P* = 0.0002) and CXCL10 (*P* = 0.0002), but not in CCL2 and miR-146a. Similarly, SLE patients with decreasing STAT1 (n = 13; *P* = 0.0001, Figure 3D) had significant decrease in IFN score (*P* = 0.0001) and CXCL10 (*P* = 0.0004), whereas no significant changes in CCL2 and miR-146a were observed. By ranking patients according to decreasing IFN score or STAT1, the reversal of the results from ranking by increasing IFN score or STAT1 should ideally have been observed. Interestingly, the exception was observed only for CCL2 and miR-146a (Figure 3C, D).

### Relationship of IFN score to other biomarkers

**Figure 4:**
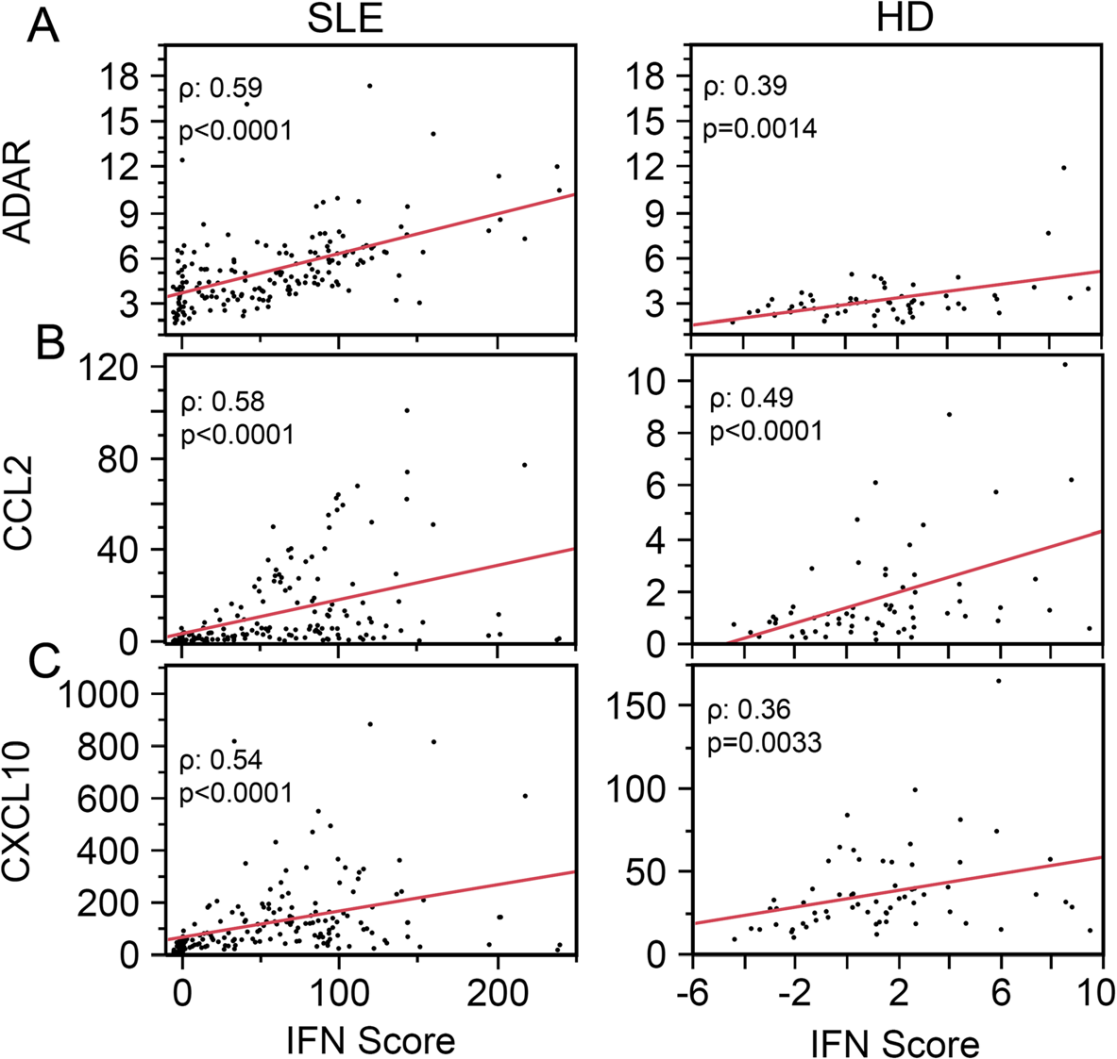
**ADAR, CCL2, and CXCL10 levels correlate with IFN score in both systemic lupus erythematosus (SLE) patients and healthy donors (HD). (A)** ADAR, **(B)** CCL2, and **(C)** CXCL10 display direct and significant *r*^2^ with IFN score for both SLE visits and HD. ADAR, adenosine deaminase acting on RNA; CCL2, C-C motif chemokine ligand 2; CXCL10, C-X-C motif chemokine 10.

**Figure 5:**
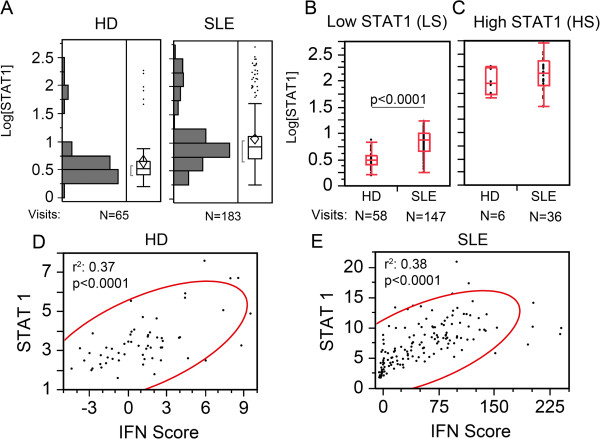
**Bimodal distribution of STAT1 into high and low groups. (A)** The log_10_ transformation of STAT1 shows a bimodal distribution of STAT1 with two populations (high- and low-STAT1 groups) with a cut off at 1.5 log[relative fold-change] of STAT1 (Log[STAT1]) for both healthy donor (HD) and SLE patient visits. **(B)** The low-STAT1 groups displayed significant difference of STAT1 in SLE patient visits compared to HD. **(C)** On the other hand, the high-STAT1 groups showed no significant difference between SLE and HD. **(D**, **E)** In the low-STAT1 group, STAT1 levels display a direct correlation to the IFN score in SLE patient visits and HD. STAT, signal transducers and activators of transcription.

To better understand whether the association of IFN score with the other biomarkers in paired patient-visits could be expanded, levels of ADAR, CCL2, and CXCL10 from the entire cohort of SLE patient visits and HD were correlated to IFN score (Figure 4). ADAR, CCL2, and CXCL10 displayed significant coefficient of determination (*r*^2^) in both SLE and HD (Figure 4). The consistent significant correlations of these genes to IFN from the low levels observed in HD (Figure 4, right panels) to aberrantly high pathogenic levels of IFN in SLE patient visits (Figure 4, left panels) was indicative of a normal intrinsic response of ADAR, CCL2, and CXCL10 to IFN production. Contrary to an earlier report showing that the level of miR-146a was negatively correlated with IFN score [46], miR-146a as well as pri-miR-146a did not display any significant correlation with IFN score in either HD or SLE patients (data not shown). Surprisingly, in the same type of analysis, STAT1 did not display a significant correlation to IFN score either (data not shown). Further analysis of STAT1 expression revealed two populations after applying a log_10_ transformation (Log[STAT1]) in both HD and SLE patients (Figure 5A). Using an arbitrary cut off of 1.50 Log[STAT1] to segregate STAT1 results, values below 1.50 were referred as the low-STAT1 group and above 1.50 were the high-STAT1 group (Figure 5B, C). In the low STAT1 group, SLE patient visits displayed significantly higher expression of STAT1 compared to HD (2.44-fold, *P* <0.0001, Figure 5B), but in the high-STAT1 group, no significant difference was observed (Figure 5C). Furthermore, the low-STAT1 group displayed significant positive association between STAT1 and IFN score in both HD (Figure 5D) and SLE patients (Figure 5E). In contrast, in the high-STAT1 group there was no correlation between STAT1 and IFN score (data not shown).

### STAT1 levels correlate with SLE activity

**Figure 6:**
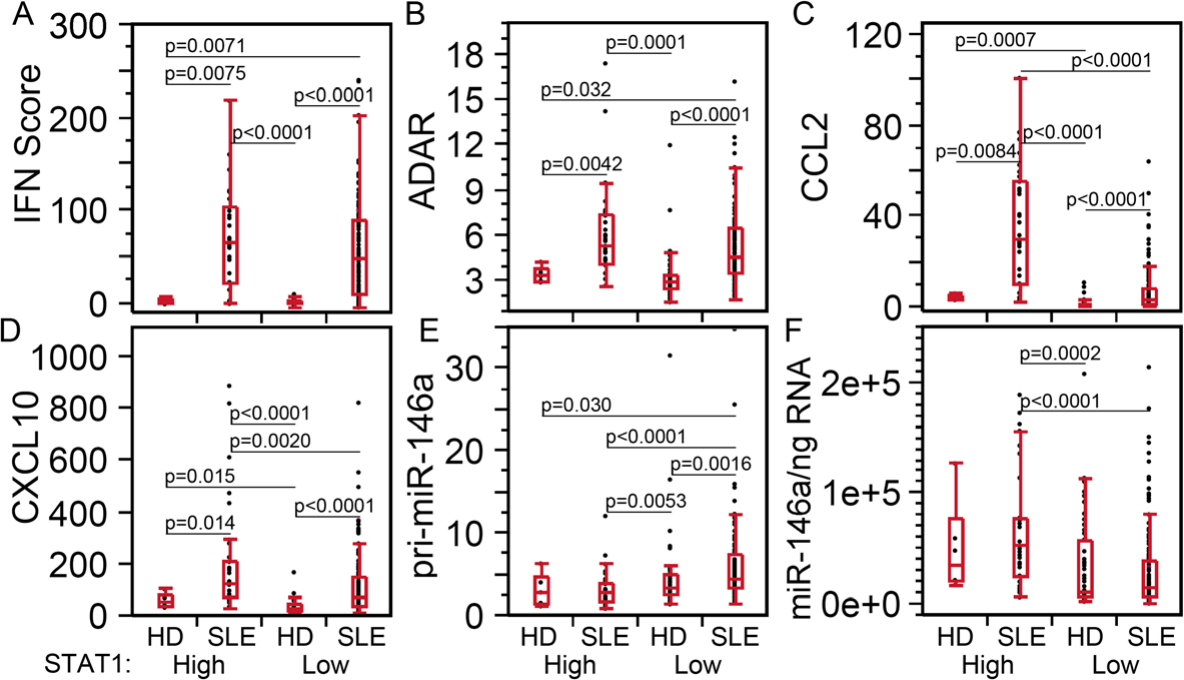
**High levels of CCL2, CXCL10, and miR-146a compared to low STAT1 in high STAT1 systemic lupus erythematosus (SLE) patients. (A)** IFN score, **(B)** ADAR, **(C)** CCL2, **(D)** CXCL10, **(E)** pri-miR-146a, and **(F)** miR-146a were compared in SLE patient visits and healthy donors (HD), which were segregated by high and low STAT1 levels demonstrating that patients with high STAT1 SLE expressed higher levels of CCL2, CXCL10, and miR-146a than those with low STAT1. STAT, signal transducers and activators of transcription; ADAR, adenosine deaminase acting on RNA; CCL2, C-C motif chemokine ligand 2; CXCL10, C-X-C motif chemokine 10.

The effects of high and low STAT1 on IFN score and ADAR appeared to be related to the active versus inactive status of SLE (SLEDAI, Figure 1A, C) and anti-dsDNA(+) versus (−) patients (Figure 2A, C) where IFN score and ADAR were significantly higher than in HD, but not significantly different between SLE patient visits with high and low STAT1 (Figure 6A, B). CCL2 was significantly different between active and inactive SLE, and between HD and active and inactive SLE as well (Figure 1D), which resembles the results of anti-dsDNA (+ versus -) (Figure 2D) and high- versus low-STAT1 comparisons (Figure 6D). Similar observations are valid for CCL2, with the addition that there is a difference in CCL2 expression between high- and low-STAT1 SLE (Figure 6C). As both SLEDAI active and anti-dsDNA (+) are indicators of increased disease activity, these results indicate that patients with high STAT1 are also in a more active disease state than those with low STAT1.

To determine whether ethnicity could be a confounding factor for the effects of high and low STAT1, IFN score, CCL2, and CXCL10 levels were segregated based on ethnicity and high and low STAT1 (Additional file 1: Figure S3A-C). Overall, high STAT1 patient visits did not show a significant difference among AA, EA, and LA. However, low-STAT1 AA patients showed significantly higher IFN score, CCL2 and CXCL10 compared to other groups (Additional file 1: Figure S3). These results indicated that high- and low-STAT1 groups were identified essentially in all ethnicities, and differences in IFN score, CCL2, and CXCL2 levels were observed among low-STAT1 groups but not among the high-STAT1 groups.

### STAT1 influences the covariance of IFN score with ADAR, CCL2, and CXCL10

**Figure 7:**
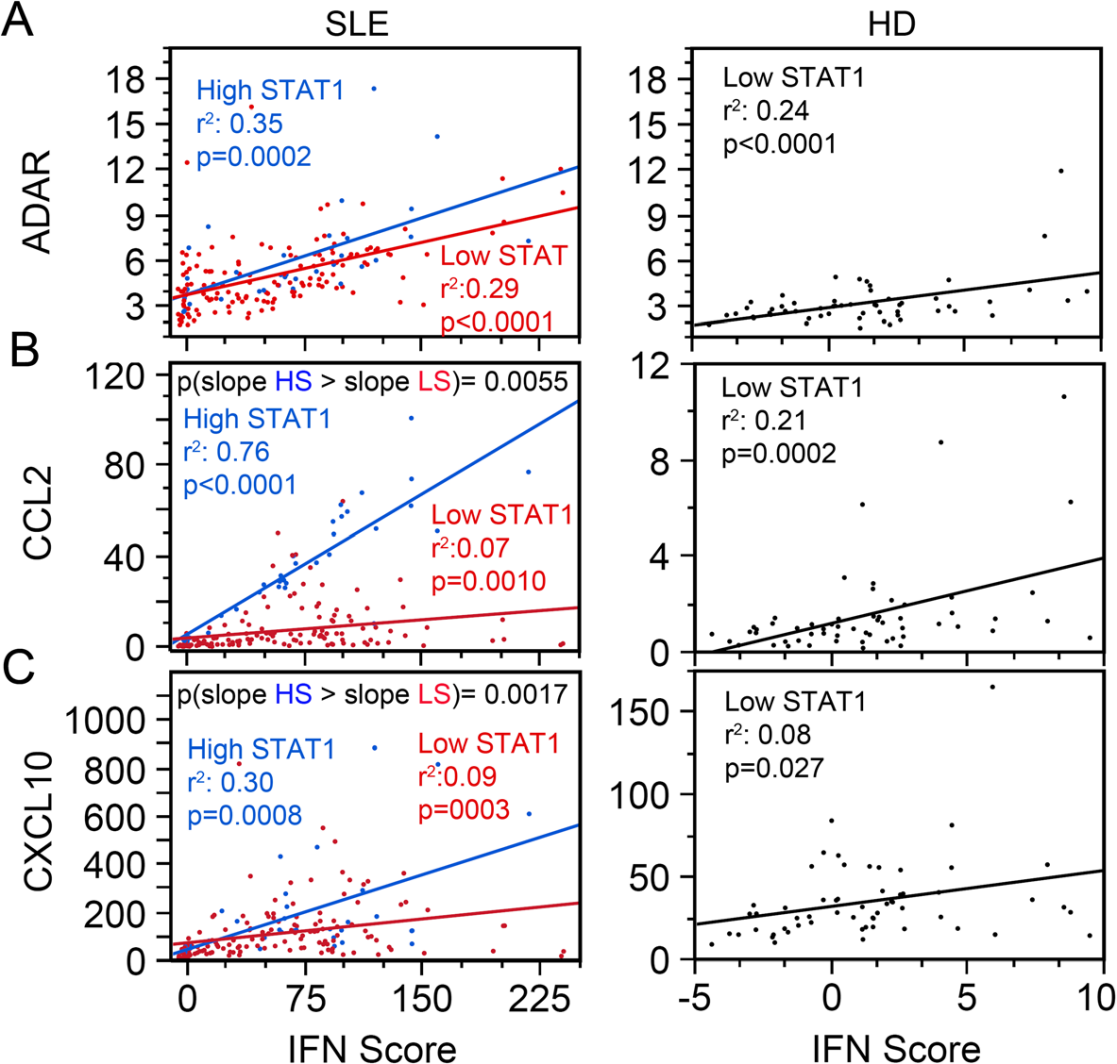
**Effect of high versus low STAT1 expression in ADAR, CCL2, and CXCL10 correlation with IFN score. (A)** ADAR, **(B)** CCL2, and **(C)** CXCL10 displayed a significant linear coefficient of determination (*r*^2^) with IFN score for high STAT1 (HS, blue, left panels). For the SLE patient visits with low STAT1 (LS, red, left panels) *r*^2^ resembled that of the LS healthy donors (HD) (black, right panels). **(B**, **C)** The slopes of high STAT1 for CCL2 and CXCL10 were significantly higher than those for patient visits with low STAT1 (left panels). ADAR, adenosine deaminase acting on RNA; CCL2, C-C motif chemokine ligand 2; CXCL10, C-X-C motif chemokine 10.

To determine whether high versus low STAT1 levels affected the correlation between IFN score and the other biomarkers, we analyzed these parameters in patients with high versus low STAT1 expression (Figure 7). Even though ADAR expression was reported to be STAT1-independent [62,63], patient visits with low-STAT1 SLE (red, *r*^2^ = 0.29, *P* <0.0001), high-STAT1 SLE (blue, *r*^2^ = 0.35, *P* = 0.0002) patients, and low-STAT1 HD (black, *r*^2^ = 0.24, *P* <0.0001) displayed significant association between ADAR and IFN score (Figure 7A). Similarly, CCL2 was significantly associated with IFN score in patient visits with low STAT1 SLE (*r*^2^ = 0.07, *P* <0.0010), patient visits with high-STAT1 SLE (*r*^2^ = 0.76, *P* <0.0001), and HD with low STAT1 (*r*^2^ = 0.08, *P* = 0.0002); also CXCL10 displayed significant association with IFN score for SLE patients with low STAT1 (*r*^2^ = 0.09, *P* = 0.0003), patient visits with high STAT1 SLE (*r*^2^ = 0.30, *P* = 0.0008), and HD with low STAT1 (*r*^2^ = 0.08, *P* = 0.027, Figure 7B, C).

The slope of the linear regression represents the rate of change of ADAR, CCL2, and CXCL10 per unit of change in IFN score. This led to the intriguing possibility that patient visits with high STAT1 have a higher slope than those with low STAT1. ANCOVA was used to test if the slopes were significantly different (Figure 7). ADAR/IFN scores were not significantly different between high- and low-STAT1 patients (Figure 7A, blue versus red line, *P*-value not shown), but CCL2/IFN score and CXCL10/IFN score slopes were significantly higher in the high-STAT1 (HS) patients compared to the low STAT1 (LS) patients (Figure 7B, C, blue versus red line). This suggests that high STAT1 levels may enhance CCL2 and CXCL10 expression potentially induced by IFN.

Next, we studied whether ethnic background could influence the association of IFN score with CCL2 and CXCL10 and altered the effects of high and low STAT1 (See Additional file 1: Figure S4). Influence of ethnic background appeared to be minimal on CCL2 in high-STAT1 patient visits. CCL2 in high-STAT1 AA, EA, and LA displayed very good linear correlation (*r*^2^ >0.59, *P* ≤0.0018) with IFN score (See Additional file 1: Figure S4A, C, E). Low-STAT1 EA and LA also showed good linear correlation (*r*^2^ ≥0.16, *P* ≤0.039, red, Additional file 1: Figure S4C, E); however, low-STAT1 AA did not display a linear correlation between CCL2 and IFN score (See Additional file 1: Figure S4A).

CXCL10 had a significant correlation (*r*^2^ >0.24, *P* ≤0.032) with IFN score for high-STAT1 AA and EA (See Additional file 1: Figure S4B, D); however, CXCL10 had significant correlation (*r*^2^ >0.25, *P* ≤0.0002) with IFN score for low-STAT1 EA and LA (See Additional file 1: Figure S4A, D, F). AsA could not ascertain significant correlations for CCL2/IFN score and CXCL10, probably due to the small sample size (data not shown).

### Induction of STAT1, CCL2, and CXCL10 in THP-1 cells with type I IFN

**Figure 8:**
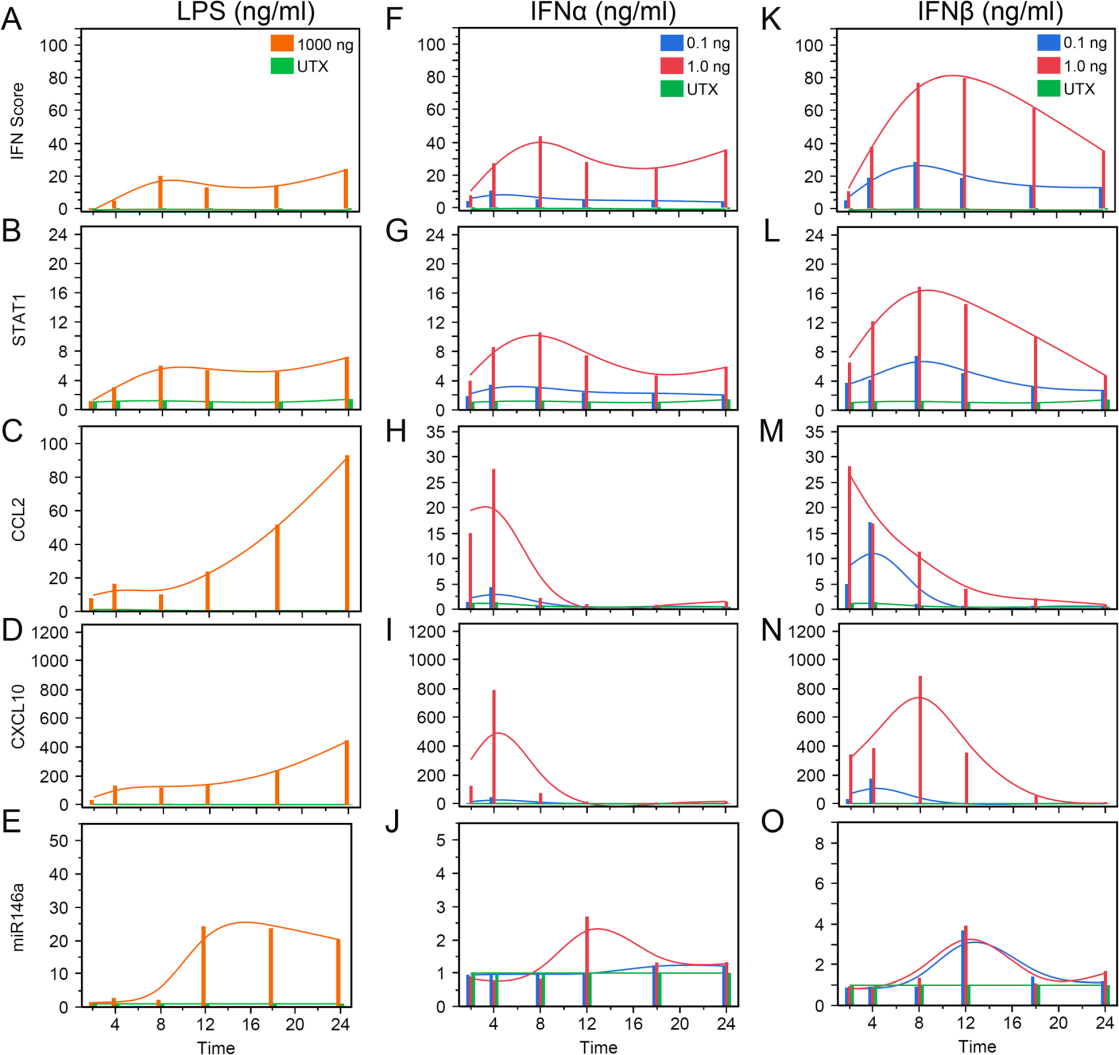
**THP-1 response to IFNα, IFNβ, and LPS over a period of 24 h.** THP-1 cells were treated with different doses of IFNα, IFNβ, and LPS and lysates were harvested at various times from 2 to 24 h for RNA isolation and analyses. IFN score **(A**, **F**, **K)**, and the expression of STAT1 **(B**, **G**, **L)**, CCL2 **(C**, **H**, **M)**, CXCL10 **(D**, **I**, **N)**, and miR-146a **(E**, **J**, **O)** were evaluated at 0.1 and 1.0 ng/ml of IFNα2 and IFNβ as well as 1,000 ng/ml of LPS. LPS, lipopolysaccharide; STAT, signal transducers and activators of transcription; CCL2, C-C motif chemokine ligand 2; CXCL10, C-X-C motif chemokine 10; UTX, untreated.

TLRs have been implicated to play a role in SLE pathogenesis. To model the response of STAT1, CCL2, and CXCL10 as well as IFN-I, TLR4 was stimulated in human monocytic THP-1 for 24 h with 1,000 ng/ml of LPS. IFN score increased at around 4 h and peaked around 8 h (Figure 8A). In 1.0 ng/ml of IFNα2-treated and 0.1 ng/ml of IFNβ-treated THP-1 cells, IFN score displayed a similar trend as in LPS treatment (Figure 8F, K); however for 1.0 ng/ml IFNβ-treated cells, IFN score increased up till 12 h (Figure 8K), whereas 0.1 ng/ml-treated cells displayed little change (Figure 8F). These results demonstrated THP-1 responsiveness to IFN-I as well as the fact that they were capable of IFN-I production.

Interestingly, whereas LPS displayed a gradual, long-term increase of CCL2 and CXCL10, IFNα2 and IFNβ treatments displayed rapid increases followed by decreases of CCL2 and CXCL10. After LPS stimulation, STAT1 did not increase till 4 h and reached its peak expression at 8 h (Figure 8B); however in THP-1 cells stimulated with IFNα2 or IFNβ, STAT1 increased at 2 h, peaking at 8 h (Figure 8G, L). CCL2 increased at 2 h in LPS-treated THP-1 cells and continued to increase during the 24-h period (Figure 8C); however this was not until after maximum expression of STAT1 was reached (Figure 8B), and CCL2 began to rapidly increase (Figure 8C). CCL2 increased at 2 h in 0.1 and 1.0 ng/ml IFNα2-treated as well as 0.1 ng/ml IFNβ-treated THP-1 cells, but it peaked at 4 h and began to decrease rapidly (Figure 8H). For 1.0 ng/ml IFNβ treatment of THP-1 cells, the peak was shifted by 2 h so that CCL2 peaked at 2 h and began to rapidly decrease (Figure 8M). CXCL10 displayed a trend similar to CCL2 for 1.0 ng/ml IFNα2-treated and 0.1 ng/ml IFNβ-treated THP-1 cells (Figure 8I, N). In 1.0 ng/ml IFNβ treatment of THP-1 cells, CXCL10 continued till 8 h (Figure 8N). These results indicated that CCL2 and CXCL10 rapidly responded to IFNα2 and IFNβ stimulation whereas TLR4 stimulation appeared to induce a slow gradual increase, but then a rapid increase after STAT1 reached its maximum expression.

miR-146a appeared to differ in its response from the other biomarkers. LPS upregulated miR-146 3-fold and it rapidly reached a peak of an 11-fold increase at 12 h (Figure 8E). miR-146a in IFNα2- or IFNβ-treated cells showed a modest of 3- to 4-fold peak at 8 h, potentially indicating that IFN-I did not induce significant production of miR-146a (Figure 8J, O).

## Discussion

In this study, expression of previously identified SLE biomarkers was examined and correlation tested with demographic and clinical parameters, focusing on the analysis of a possible correlation among them. The primary analyses used ordinary linear regression, even for data from multiple visits, as reported in Figures 4, 5, and 7. Alternatively, the GEE model for repeated measures was also used to account for possible within-subject effects from patients with multiple visits [54]. When we compared the parameters (slope and significance) from the GEE and ordinary linear regression, the results were practically identical (data not shown). It is known that unless the vast majority of the samples have repeated measures (patients with multiple visits), the ordinary linear regression is expected to closely approximate the GEE model [64]. Furthermore, even if there was strong correlation between visits of patients, ordinary linear regression would underestimate the correlation because it assumes that the visits are independent; therefore, the correlations of ordinary linear regressions are more stringent than those of GEE [64]. In addition, we also assessed the normality of each dataset before applying linear regression. With the exception of STAT1, the IFN score, ADAR, CCL2, and CXCL10 resembled normal distributions (data not shown). In most cases when dealing with such large datasets, even moderate deviations from normalcy are not critical due to the central limit theorem [65]. For these reasons, we decided to report ordinary linear regression rather than the more complex GEE model for repeated samples.

### Biomarker assessment

Our results show that ADAR, STAT1, CCL2, and CXCL10 levels were significantly elevated in the SLE cohort as expected. This is in part validated by previously published results showing increased levels of these biomarkers and their correlation to IFN-I production in SLE patients [1,2,6,21,22,66]. Furthermore, our study shows that THP-1 cells treated with IFNα2 or IFNβ display up to 18-fold increase of STAT1, 25-fold increase of CCL2, and 700-fold increase of CXCL10, confirming that these genes respond to IFN-I stimulation.

Tang *et al*. reported miR-146a under-expression in SLE PBMCs [46], whereas we did not observe a decrease or a difference between patients with active or inactive SLE for miR-146a expression in peripheral blood leukocytes of SLE patients in our cohort. Luo *et al*. [67] hypothesize that a functional variant in the miR-146a promoter may be responsible for decreased levels of miR-146a in SLE, so the pri-miR-146a levels should be decreased in our population; however, no significant differences in pri-miR-146a expression were observed in our population. Furthermore, Tang *et al*. reported inverse correlation between miR-146a and IFN score in their SLE cohort, while we did not observe a significant correlation in our cohort. A significant increase in miR-146a was observed only in SLE patients with increasing IFN score between the initial and the second visit [36]. Other possible explanations for the discrepancy between the two datasets could be the difference in cell populations and racial composition in our cohort versus the one examined by Tang *et al*. and Luo *et al*. [46,67]. As for the THP-1 monocyte cell model, IFN-I weakly stimulated miR-146a expression compared to LPS. All these results suggest that the role of miR-146a in regulating IFN-I in our cohort of SLE patients may have been limited.

### Biomarker connections

Previous reports have demonstrated the involvement of ADAR mRNA and CCL2 and CXCL10 protein in SLE [15-17,22]. In published literature, ADAR mRNA and CCL2, CXCL10 protein levels displayed a positive association with IFN score [11,21,22,68]. Similarly in our cohort, directly correlation between IFN score and mRNA levels of ADAR, CCL2, and CXCL10 was observed. This was observed not only in SLE but in HD as well, potentially indicating that these genes are responding normally to IFN even when at levels aberrantly elevated. Unlike reports from previous studies, STAT1 did not correlate well with the IFN score in the SLE patient population [69,70]. Instead, patients with low-STAT1 SLE and HD with low-STAT1, the expression was associated with IFN score. Patients paired by two visits that were ranked by increasing IFN score demonstrate strong covariance with STAT1, but the covariance between IFN score and increasing STAT1 appeared to be weaker. In paired SLE patient visits, decreasing IFN scores or STAT1 level is accompanied by a decrease of the other biomarkers suggesting that STAT1 and IFN-I may be driving factors.

When SLE patient visits are grouped into high and low STAT1, high-STAT1 SLE patient visits showed significantly higher levels of CCL2 and CXCL10. After grouping by high and low STAT1, the high-STAT1 patient visits showed a significantly increased slope for CCL2/IFN and CXCL10/IFN scores compared to low-STAT1 SLE patient visits. This enhanced response by CCL2 and CXCL10 to IFN-I in high-STAT1 patients may be due in part to the role of STAT1 in activation of CCL2 and CXCL10 [71-73]. Hence, STAT1 levels appear to be enhancing chemokine response to IFN-I.

Furthermore, THP-1 cells treated with IFNα2, IFNβ, or even LPS, demonstrated that IFN score, STAT1, CCL2, CXCL10 and miR-146a were upregulated in a time-dependent manner. IFNα2 or IFNβ treatment of THP-1 cells shows that cells expressed decreased levels of CCL2 and CXCL10 shortly after reaching their peak expression, whereas LPS treatment displayed a steady increase of CCL2 and CXCL10 with a less rapid induction compared to their expression after IFNα2 or IFNβ stimulation. After STAT1 peak expression in LPS-treated THP-1 cells, CCL2 and CXCL10 expression rapidly accelerated. On the contrary, IFNα2 and IFNβ treatment of THP-1 cells shows that CCL2 and CXCL10 both started decreasing after reaching their peak expression, whereas STAT1 continued to increase with IFNα2 or IFNβ stimulation. These results indicate that CCL2 and CXCL10 respond differently to TLR4 stimulation compared to IFN signaling. It also indicates that CCL2 and CXCL10 response to IFN-I is rapid but short compared to TLR signaling, as IFN score correlates with greater increase of CCL2 and CXCL10 in the high-STAT1 patients than in low-STAT1 patients. The results of TLR4 stimulation suggest that at least in the high-STAT1 patient population CCL2 and CXCL10 are being driven by TLR signaling rather than IFN-I directly since IFN-I stimulation caused a rapid increase followed by an equally rapid decrease of CCL2 and CXCL10 independent of STAT1 expression.

It is unclear why STAT1 was elevated to such high levels in some of the SLE patients and HD. One possibility is from TLR activation as seen in the LPS stimulations. Another possibility is impairment in the expression of miR-146a, which is known to target STAT1 [46]. In the paired SLE-patient visits, miR-146a might be increased as a response to STAT1 increases, but it is unable to downregulate STAT1. One potential reason that miR-146a is unable to downregulate STAT1 is due to alternative splicing. STAT1 exists as a long form (STAT1a) and short form (STAT1b). According to the miRNA target prediction site, TargetScan.com, STAT1b has a shorter 3′ UTR compared to STAT1a 3′ UTR. The shorter 3′ UTR in STAT1b lacks miR-146a binding sites, which would prevent miR-146a downregulation of STAT1b. Several HD also displayed very high STAT1 levels, however CCL2 and CXCL10, even though elevated compared to low-STAT1 HD, were significantly lower than in SLE patients. A potential reason is that IFN-I drives CCL2 and CXCL10 expression, and high STAT1 primes the immune system to amplify CCL2 and CXCL10 expression when IFN-I is present. Without IFN-I, the high STAT1 levels may still prime the immune system but they lack the ignition to drive the process forward.

## Conclusions

The results of this study show that STAT1 mRNA expression in PBMCs from lupus patients and healthy controls is segregated into high- or low-STAT1 groups. STAT1 may be an important driver of lupus pathogenesis with STAT1 serving as an expression enhancer of CCL2 and CXCL10 in patients with high levels of STAT1.

## Abbreviations

AA: African Americans; ACR: American College of Rheumatology; ADAR: adenosine deaminase acting on RNA; ANA: antinuclear antibody; ANCOVA: analysis of covariance; AsA: Asian Americans; BSA: bovine serum albumin; CCL2: C-C motif chemokine ligand 2; CXCL10: C-X-C motif chemokine 10; dsDNA: double-stranded DNA; dsRNA: double-stranded RNA; EA: European Americans; ELISA: enzyme-linked immunosorbent assay; FBS: fetal bovine serum; HD: healthy donors; IFNAR: interferon receptor; IFN-I: type I interferon; IgG: immunoglobulin G; IrA: interracial Americans; IRF: interferon regulatory factor; LA: Latin Americans; LPS: lipopolysaccharide; LY6E: lymphocyte antigen 6 complex locus E; miRNA: microRNA; MX1: myxovirus resistance 1; OAS1: 2′,5′-oligoadenylate synthetase; OD: optical density; PBMC: peripheral blood mononuclear cells; PBS: phosphate-buffered saline; PKR: protein kinase RNA-activated; SLE: systemic lupus erythematosus; SLEDAI: systemic lupus erythematosus disease activity index; STAT: signal transducers and activators of transcription; TLR: toll-like receptor; TNF: tumor necrosis factor; UTR: untranslated region.

## Competing interests

The authors declare that they have no competing interests.

## Authors’ contributions

PRDG carried out the experiments. PRDG, MS and EKLC designed the study. PRDG, AC, and MS performed the statistical analysis. ESS, AC, and WHR enrolled patients for the study, collected information and maintained the database. PRDG, AC, and EKLC drafted the manuscript. All authors read and approved the final manuscript.

## Supplementary Material

Additional file 1: Figure S1 Anti-dsDNA level, IFN score, STAT1, CCL2, and CXCL10 in individuals with different ethnic backgrounds. **Figure S2**. Comparison of SLEDAI, anti-dsDNA titer, IFN score, STAT1, CCL2, and CXCL10 in patients with different ethnic background. **Figure S3**. IFN score, CCL2, and CXCL10 in individuals with different ethnic background and STAT1 levels. **Figure S4**. CCL2, CXCL10, and IFN score in individuals with different ethnic background and high vs low STAT1 groups. dsDNA, double-stranded DNA; STAT, signal transducers and activators of transcription; CCL2, C-C motif chemokine ligand 2; CXCL10, C-X-C motif chemokine 10; SLEDAI, systemic lupus erythematosus disease activity index. Click here for file

## References

[B1] Baechler EC Batliwalla FM Karypis G Gaffney PM Ortmann WA Espe KJ Shark KB Grande WJ Hughes KM Kapur V Gregersen PK Behrens TW Interferon-inducible gene expression signature in peripheral blood cells of patients with severe lupus Proc Natl Acad Sci USA 2003 16 2610 2615 12604793 10.1073/pnas.0337679100 PMC151388

[B2] Bennett L Palucka AK Arce E Cantrell V Borvak J Banchereau J Pascual V Interferon and granulopoiesis signatures in systemic lupus erythematosus blood J Exp Med 2003 16 711 723 12642603 10.1084/jem.20021553 PMC2193846

[B3] Crow MK Interferon pathway activation in systemic lupus erythematosus Curr Rheumatol Rep 2005 16 463 468 16303107 10.1007/s11926-005-0053-4

[B4] Preble OT Black RJ Friedman RM Klippel JH Vilcek J Systemic lupus erythematosus: presence in human serum of an unusual acid-labile leukocyte interferon Science 1982 16 429 431 6176024 10.1126/science.6176024

[B5] Nikpour M Dempsey AA Urowitz MB Gladman DD Barnes DA Association of a gene expression profile from whole blood with disease activity in systemic lupus erythaematosus Ann Rheum Dis 2008 16 1069 1075 18063674 10.1136/ard.2007.074765

[B6] Crow MK Kirou KA Wohlgemuth J Microarray analysis of interferon-regulated genes in SLE Autoimmunity 2003 16 481 490 14984025 10.1080/08916930310001625952

[B7] Kirou KA Lee C George S Louca K Papagiannis IG Peterson MG Ly N Woodward RN Fry KE Lau AY Prentice JG Wohlgemuth JG Crow MK Coordinate overexpression of interferon-alpha-induced genes in systemic lupus erythematosus Arthritis Rheum 2004 16 3958 3967 15593221 10.1002/art.20798

[B8] Bass BL Weintraub H An unwinding activity that covalently modifies its double-stranded RNA substrate Cell 1988 16 1089 1098 3203381 10.1016/0092-8674(88)90253-x

[B9] Wagner RW Smith JE Cooperman BS Nishikura K A double-stranded RNA unwinding activity introduces structural alterations by means of adenosine to inosine conversions in mammalian cells and Xenopus eggs Proc Natl Acad Sci USA 1989 16 2647 2651 2704740 10.1073/pnas.86.8.2647 PMC286974

[B10] Nishikura K Functions and regulation of RNA editing by ADAR deaminases Annu Rev Biochem 2010 16 321 349 20192758 10.1146/annurev-biochem-060208-105251 PMC2953425

[B11] George CX Gan Z Liu Y Samuel CE Adenosine deaminases acting on RNA, RNA editing, and interferon action J Interferon Cytokine Res 2011 16 99 117 21182352 10.1089/jir.2010.0097 PMC3034097

[B12] Nie Y Hammond GL Yang JH Double-stranded RNA deaminase ADAR1 increases host susceptibility to virus infection J Virol 2007 16 917 923 17079286 10.1128/JVI.01527-06 PMC1797455

[B13] Toth AM Li Z Cattaneo R Samuel CE RNA-specific adenosine deaminase ADAR1 suppresses measles virus-induced apoptosis and activation of protein kinase PKR J Biol Chem 2009 16 29350 29356 19710021 10.1074/jbc.M109.045146 PMC2785566

[B14] Li Z Wolff KC Samuel CE RNA adenosine deaminase ADAR1 deficiency leads to increased activation of protein kinase PKR and reduced vesicular stomatitis virus growth following interferon treatment Virology 2010 16 316 322 19913273 10.1016/j.virol.2009.10.026 PMC2789878

[B15] Laxminarayana D Khan IU Kammer G Transcript mutations of the alpha regulatory subunit of protein kinase A and up-regulation of the RNA-editing gene transcript in lupus T lymphocytes Lancet 2002 16 842 849 12243919 10.1016/s0140-6736(02)09966-x

[B16] Laxminarayana D O’Rourke KS Maas S Olorenshaw I Altered editing in RNA editing adenosine deaminase ADAR2 gene transcripts of systemic lupus erythematosus T lymphocytes Immunology 2007 16 359 369 17376196 10.1111/j.1365-2567.2007.02582.x PMC2265949

[B17] Orlowski RJ O’Rourke KS Olorenshaw I Hawkins GA Maas S Laxminarayana D Altered editing in cyclic nucleotide phosphodiesterase 8A1 gene transcripts of systemic lupus erythematosus T lymphocytes Immunology 2008 16 408 419 18462347 10.1111/j.1365-2567.2008.02850.x PMC2669144

[B18] Karonitsch T Feierl E Steiner CW Dalwigk K Korb A Binder N Rapp A Steiner G Scheinecker C Smolen J Aringer M Activation of the interferon-gamma signaling pathway in systemic lupus erythematosus peripheral blood mononuclear cells Arthritis Rheum 2009 16 1463 1471 19404947 10.1002/art.24449

[B19] Usacheva A Smith R Minshall R Baida G Seng S Croze E Colamonici O The WD motif-containing protein receptor for activated protein kinase C (RACK1) is required for recruitment and activation of signal transducer and activator of transcription 1 through the type I interferon receptor J Biol Chem 2001 16 22948 22953 11301323 10.1074/jbc.M100087200

[B20] Ivashkiv LB Type I interferon modulation of cellular responses to cytokines and infectious pathogens: potential role in SLE pathogenesis Autoimmunity 2003 16 473 479 14984024 10.1080/08916930310001605882

[B21] Bauer JW Baechler EC Petri M Batliwalla FM Crawford D Ortmann WA Espe KJ Li W Patel DD Gregersen PK Behrens TW Elevated serum levels of interferon-regulated chemokines are biomarkers for active human systemic lupus erythematosus PLoS Med 2006 16 e491 17177599 10.1371/journal.pmed.0030491 PMC1702557

[B22] Bauer JW Petri M Batliwalla FM Koeuth T Wilson J Slattery C Panoskaltsis-Mortari A Gregersen PK Behrens TW Baechler EC Interferon-regulated chemokines as biomarkers of systemic lupus erythematosus disease activity: a validation study Arthritis Rheum 2009 16 3098 3107 19790071 10.1002/art.24803 PMC2842939

[B23] Yadav A Saini V Arora S MCP-1: chemoattractant with a role beyond immunity: a review Clin Chim Acta 2010 16 1570 1579 20633546 10.1016/j.cca.2010.07.006

[B24] La Gruta NL Kedzierska K Stambas J Doherty PC A question of self-preservation: immunopathology in influenza virus infection Immunol Cell Biol 2007 16 85 92 17213831 10.1038/sj.icb.7100026

[B25] Marsche G Semlitsch M Hammer A Frank S Weigle B Demling N Schmidt K Windischhofer W Waeg G Sattler W Malle E Hypochlorite-modified albumin colocalizes with RAGE in the artery wall and promotes MCP-1 expression via the RAGE-Erk1/2 MAP-kinase pathway FASEB J 2007 16 1145 1152 17218539 10.1096/fj.06-7439com PMC4864469

[B26] Lloyd CM Minto AW Dorf ME Proudfoot A Wells TN Salant DJ Gutierrez-Ramos JC RANTES and monocyte chemoattractant protein-1 (MCP-1) play an important role in the inflammatory phase of crescentic nephritis, but only MCP-1 is involved in crescent formation and interstitial fibrosis J Exp Med 1997 16 1371 1380 9104823 10.1084/jem.185.7.1371 PMC2196251

[B27] Kulkarni O Pawar RD Purschke W Eulberg D Selve N Buchner K Ninichuk V Segerer S Vielhauer V Klussmann S Anders HJ Spiegelmer inhibition of CCL2/MCP-1 ameliorates lupus nephritis in MRL-(Fas)lpr mice J Am Soc Nephrol 2007 16 2350 2358 17625118 10.1681/ASN.2006121348

[B28] Dufour JH Dziejman M Liu MT Leung JH Lane TE Luster AD IFN-gamma-inducible protein 10 (IP-10; CXCL10)-deficient mice reveal a role for IP-10 in effector T cell generation and trafficking J Immunol 2002 16 3195 3204 11907072 10.4049/jimmunol.168.7.3195

[B29] Luster AD Jhanwar SC Chaganti RS Kersey JH Ravetch JV Interferon-inducible gene maps to a chromosomal band associated with a (4;11) translocation in acute leukemia cells Proc Natl Acad Sci USA 1987 16 2868 2871 2437586 10.1073/pnas.84.9.2868 PMC304761

[B30] Loetscher M Loetscher P Brass N Meese E Moser B Lymphocyte-specific chemokine receptor CXCR3: regulation, chemokine binding and gene localization Eur J Immunol 1998 16 3696 3705 9842912 10.1002/(SICI)1521-4141(199811)28:11<3696::AID-IMMU3696>3.0.CO;2-W

[B31] Weng Y Siciliano SJ Waldburger KE Sirotina-Meisher A Staruch MJ Daugherty BL Gould SL Springer MS DeMartino JA Binding and functional properties of recombinant and endogenous CXCR3 chemokine receptors J Biol Chem 1998 16 18288 18291 9660793 10.1074/jbc.273.29.18288

[B32] Han C Fu J Liu Z Huang H Luo L Yin Z Dipyrithione inhibits IFN-gamma-induced JAK/STAT1 signaling pathway activation and IP-10/CXCL10 expression in RAW264.7 cells Inflamm Res 2010 16 809 816 20372968 10.1007/s00011-010-0192-6 PMC7079753

[B33] Romagnani P Crescioli C CXCL10: a candidate biomarker in transplantation Clin Chim Acta 2012 16 1364 1373 22366165 10.1016/j.cca.2012.02.009

[B34] Liu M Guo S Hibbert JM Jain V Singh N Wilson NO Stiles JK CXCL10/IP-10 in infectious diseases pathogenesis and potential therapeutic implications Cytokine Growth Factor Rev 2011 16 121 130 21802343 10.1016/j.cytogfr.2011.06.001 PMC3203691

[B35] Nahid MA Satoh M Chan EKL MicroRNA in TLR signaling and endotoxin tolerance Cell Mol Immunol 2011 16 388 403 21822296 10.1038/cmi.2011.26 PMC3618661

[B36] Taganov KD Boldin MP Chang KJ Baltimore D NF-kappaB-dependent induction of microRNA miR-146, an inhibitor targeted to signaling proteins of innate immune responses Proc Natl Acad Sci USA 2006 16 12481 12486 16885212 10.1073/pnas.0605298103 PMC1567904

[B37] Nahid MA Rivera M Lucas A Chan EK Kesavalu L Polymicrobial infection with periodontal pathogens specifically enhances microRNA miR-146a in ApoE−/− mice during experimental periodontal disease Infect Immun 2011 16 1597 1605 21263019 10.1128/IAI.01062-10 PMC3067556

[B38] Nahid MA Satoh M Chan EKL Mechanistic role of microRNA-146a in endotoxin-induced differential cross-regulation of TLR signaling J Immunol 2011 16 1723 1734 21178010 10.4049/jimmunol.1002311 PMC3608687

[B39] Nahid MA Pauley KM Satoh M Chan EKL **miR**-**146a is critical for endotoxin**-**induced tolerance**: **Implication in innate immunity** J Biol Chem 2009 16 34590 34599 19840932 10.1074/jbc.M109.056317 PMC2787321

[B40] Pauley KM Stewart CM Gauna AE Dupre LC Kuklani R Chan AL Pauley BA Reeves WH Chan EK Cha S Altered miR-146a expression in Sjogren’s syndrome and its functional role in innate immunity Eur J Immunol 2011 16 2029 2039 21469088 10.1002/eji.201040757 PMC3760391

[B41] Sonkoly E Stahle M Pivarcsi A MicroRNAs: novel regulators in skin inflammation Clin Exp Dermatol 2008 16 312 315 18419608 10.1111/j.1365-2230.2008.02804.x

[B42] Sonkoly E Wei T Janson PC Saaf A Lundeberg L Tengvall-Linder M Norstedt G Alenius H Homey B Scheynius A Stahle M Pivarcsi A MicroRNAs: novel regulators involved in the pathogenesis of psoriasis? PLoS One 2007 16 e610 17622355 10.1371/journal.pone.0000610 PMC1905940

[B43] Nakasa T Miyaki S Okubo A Hashimoto M Nishida K Ochi M Asahara H Expression of microRNA-146 in rheumatoid arthritis synovial tissue Arthritis Rheum 2008 16 1284 1292 18438844 10.1002/art.23429 PMC2749927

[B44] Pauley KM Satoh M Chan AL Bubb MR Reeves WH Chan EKL Upregulated miR-146a expression in peripheral blood mononuclear cells from rheumatoid arthritis patients Arthritis Res Ther 2008 16 R101 18759964 10.1186/ar2493 PMC2575615

[B45] Stanczyk J Pedrioli DM Brentano F Sanchez-Pernaute O Kolling C Gay RE Detmar M Gay S Kyburz D Altered expression of MicroRNA in synovial fibroblasts and synovial tissue in rheumatoid arthritis Arthritis Rheum 2008 16 1001 1009 18383392 10.1002/art.23386

[B46] Tang Y Luo X Cui H Ni X Yuan M Guo Y Huang X Zhou H de Vries N Tak PP Chen S Shen N MicroRNA-146A contributes to abnormal activation of the type I interferon pathway in human lupus by targeting the key signaling proteins Arthritis Rheum 2009 16 1065 1075 19333922 10.1002/art.24436

[B47] Tan EM Cohen AS Fries JF Masi AT McShane DJ Rothfield NF Schaller JG Talal N Winchester RJ The 1982 revised criteria for the classification of systemic lupus erythematosus Arthritis Rheum 1982 16 1271 1277 7138600 10.1002/art.1780251101

[B48] Yamasaki Y Narain S Hernandez L Barker T Ikeda K Segal MS Richards HB Chan EK Reeves WH Satoh M Autoantibodies against the replication protein A complex in systemic lupus erythematosus and other autoimmune diseases Arthritis Res Ther 2006 16 R111 16846524 10.1186/ar2000 PMC1779422

[B49] Feng X Wu H Grossman JM Hanvivadhanakul P FitzGerald JD Park GS Dong X Chen W Kim MH Weng HH Furst DE Gorn A McMahon M Taylor M Brahn E Hahn BH Tsao BP Association of increased interferon-inducible gene expression with disease activity and lupus nephritis in patients with systemic lupus erythematosus Arthritis Rheum 2006 16 2951 2962 16947629 10.1002/art.22044

[B50] Bombardier C Gladman DD Urowitz MB Caron D Chang CH Derivation of the SLEDAI. A disease activity index for lupus patients. The Committee on Prognosis Studies in SLE Arthritis Rheum 1992 16 630 640 1599520 10.1002/art.1780350606

[B51] Touma Z Gladman DD Urowitz MB Wallace D, Hahn BH Cinical Meausres, Metrics, and indices Dubois’ Lupus Erythematosus and Related Syndromes 2012 8 Philadelphia, PA: Elsevier Health Sciences 563 581

[B52] Nahid MA Yao B Dominguez-Gutierrez PR Kesavalu L Satoh M Chan EKL Regulation of TLR2-Mediated Tolerance and Cross-Tolerance through IRAK4 Modulation by miR-132 and miR-212 J Immunol 2013 16 1250 1263 23264652 10.4049/jimmunol.1103060 PMC3552145

[B53] Livak KJ Schmittgen TD Analysis of relative gene expression data using real-time quantitative PCR and the 2(−Delta Delta C(T)) Method Methods 2001 16 402 408 11846609 10.1006/meth.2001.1262

[B54] Hanley JA Negassa A Edwardes MD Forrester JE Statistical analysis of correlated data using generalized estimating equations: an orientation Am J Epidemiol 2003 16 364 375 12578807 10.1093/aje/kwf215

[B55] Ho A Magder LS Barr SG Petri M Decreases in anti-double-stranded DNA levels are associated with concurrent flares in patients with systemic lupus erythematosus Arthritis Rheum 2001 16 2342 2349 11665975 10.1002/1529-0131(200110)44:10<2342::aid-art397>3.0.co;2-8

[B56] Bootsma H Spronk P Derksen R de Boer G Wolters-Dicke H Hermans J Limburg P Gmelig-Meyling F Kater L Kallenberg C Prevention of relapses in systemic lupus erythematosus Lancet 1995 16 1595 1599 7783536 10.1016/s0140-6736(95)90114-0

[B57] ter Borg EJ Horst G Hummel EJ Limburg PC Kallenberg CG Measurement of increases in anti-double-stranded DNA antibody levels as a predictor of disease exacerbation in systemic lupus erythematosus. A long-term, prospective study Arthritis Rheum 1990 16 634 643 2346519 10.1002/art.1780330505

[B58] Isenberg DA Garton M Reichlin MW Reichlin M Long-term follow-up of autoantibody profiles in black female lupus patients and clinical comparison with Caucasian and Asian patients Br J Rheumatol 1997 16 229 233 9133936 10.1093/rheumatology/36.2.229

[B59] Bengtsson AA Sturfelt G Truedsson L Blomberg J Alm G Vallin H Ronnblom L Activation of type I interferon system in systemic lupus erythematosus correlates with disease activity but not with antiretroviral antibodies Lupus 2000 16 664 671 11199920 10.1191/096120300674499064

[B60] Ching KH Burbelo PD Tipton C Wei C Petri M Sanz I Iadarola MJ Two major autoantibody clusters in systemic lupus erythematosus PLoS One 2012 16 e32001 22363785 10.1371/journal.pone.0032001 PMC3283706

[B61] To CH Petri M Is antibody clustering predictive of clinical subsets and damage in systemic lupus erythematosus? Arthritis Rheum 2005 16 4003 4010 16320348 10.1002/art.21414

[B62] Umareddy I Tang KF Vasudevan SG Devi S Hibberd ML Gu F Dengue virus regulates type I interferon signalling in a strain-dependent manner in human cell lines J Gen Virol 2008 16 3052 3062 19008393 10.1099/vir.0.2008/001594-0

[B63] Perry ST Buck MD Lada SM Schindler C Shresta S STAT2 mediates innate immunity to Dengue virus in the absence of STAT1 via the type I interferon receptor PLoS Pathog 2011 16 e1001297 21379341 10.1371/journal.ppat.1001297 PMC3040673

[B64] Walker GA Shostak J Common statistical methods for clinical research with SAS examples 2010 3 Cary, NC: SAS Institute Inc.

[B65] Sainani KL Dealing with non-normal data PM R 2012 16 1001 1005 23245662 10.1016/j.pmrj.2012.10.013

[B66] Qing X Putterman C Gene expression profiling in the study of the pathogenesis of systemic lupus erythematosus Autoimmun Rev 2004 16 505 509 15546798 10.1016/j.autrev.2004.07.001

[B67] Luo X Yang W Ye DQ Cui H Zhang Y Hirankarn N Qian X Tang Y Lau YL de Vries N Tak PP Tsao BP Shen N A functional variant in MicroRNA-146a promoter modulates its expression and confers disease risk for systemic lupus erythematosus PLoS Genet 2011 16 e1002128 21738483 10.1371/journal.pgen.1002128 PMC3128113

[B68] George CX Das S Samuel CE Organization of the mouse RNA-specific adenosine deaminase Adar1 gene 5′-region and demonstration of STAT1-independent, STAT2-dependent transcriptional activation by interferon Virology 2008 16 338 343 18774582 10.1016/j.virol.2008.07.029 PMC2628478

[B69] Tassiulas I Hu X Ho H Kashyap Y Paik P Hu Y Lowell CA Ivashkiv LB Amplification of IFN-alpha-induced STAT1 activation and inflammatory function by Syk and ITAM-containing adaptors Nat Immunol 2004 16 1181 1189 15467722 10.1038/ni1126

[B70] Hu X Herrero C Li WP Antoniv TT Falck-Pedersen E Koch AE Woods JM Haines GK Ivashkiv LB Sensitization of IFN-gamma Jak-STAT signaling during macrophage activation Nat Immunol 2002 16 859 866 12172544 10.1038/ni828

[B71] Fulkerson PC Zimmermann N Hassman LM Finkelman FD Rothenberg ME Pulmonary chemokine expression is coordinately regulated by STAT1, STAT6, and IFN-gamma J Immunol 2004 16 7565 7574 15585884 10.4049/jimmunol.173.12.7565

[B72] Kok SH Hong CY Kuo MY Wang CC Hou KL Lin YT Galson DL Lin SK Oncostatin M-induced CCL2 transcription in osteoblastic cells is mediated by multiple levels of STAT-1 and STAT-3 signaling: an implication for the pathogenesis of arthritis Arthritis Rheum 2009 16 1451 1462 19404962 10.1002/art.24452

[B73] Valente AJ Xie JF Abramova MA Wenzel UO Abboud HE Graves DT A complex element regulates IFN-gamma-stimulated monocyte chemoattractant protein-1 gene transcription J Immunol 1998 16 3719 3728 9759897

