# Supplementary material for: Elevated signal transducers and activators of transcription 1 correlates with increased C-C motif chemokine ligand 2 and C-X-C motif chemokine 10 levels in peripheral blood of patients with systemic lupus erythematosus
Source: Arthritis Res Ther. 2014 Jan 23;16(1):R20. doi: 10.1186/ar4448 (PMC3978614; doi:10.1186/ar4448)
Supplement: Additional file 1: Figure S1 — Anti-dsDNA level, IFN score, STAT1, CCL2, and CXCL10 in individuals with different ethnic backgrounds. Figure S2. Comparison of SLEDAI, anti-dsDNA titer, IFN score, STAT1, CCL2, and CXCL10 in patients with different ethnic background. Figure S3. IFN score, CCL2, and CXCL10 in individuals with different ethnic background and STAT1 levels. Figure S4. CCL2, CXCL10, and IFN score in individuals with different ethnic background and high vs low STAT1 groups. dsDNA, double-stranded DNA; STAT, signal transducers and activators of transcription; CCL2, C-C motif chemokine ligand 2; CXCL10, C-X-C motif chemokine 10; SLEDAI, systemic lupus erythematosus disease activity index. [file ar4448-S1.pdf]

Elevated signal transducers and activators of transcription 1 correlates with increased C-C motif chemokine ligand 2 and C-X-C motif chemokine 10 levels in peripheral blood of patients with systemic lupus erythematosus

Paul R. Dominguez-Gutierrez[1], Angela Ceribelli[1], Minoru Satoh[2,3], Eric S. Sobel[2], Westley H. Reeves[2] and Edward K.L. Chan[1*]

[1]Department of Oral Biology, University of Florida, P.O. Box 100424, 1395 Center Drive, Gainesville, FL 32610-0424, USA, [22]Division of Rheumatology and Clinical Immunology, Department of Medicine, [3]Department of Pathology, Immunology, and Laboratory Medicine, University of Florida, P.O. Box 100221, 1600 SW Archer Rd, Gainesville, FL 32610-0221, USA

Additional file 1
Figure S1-4

Figure S1

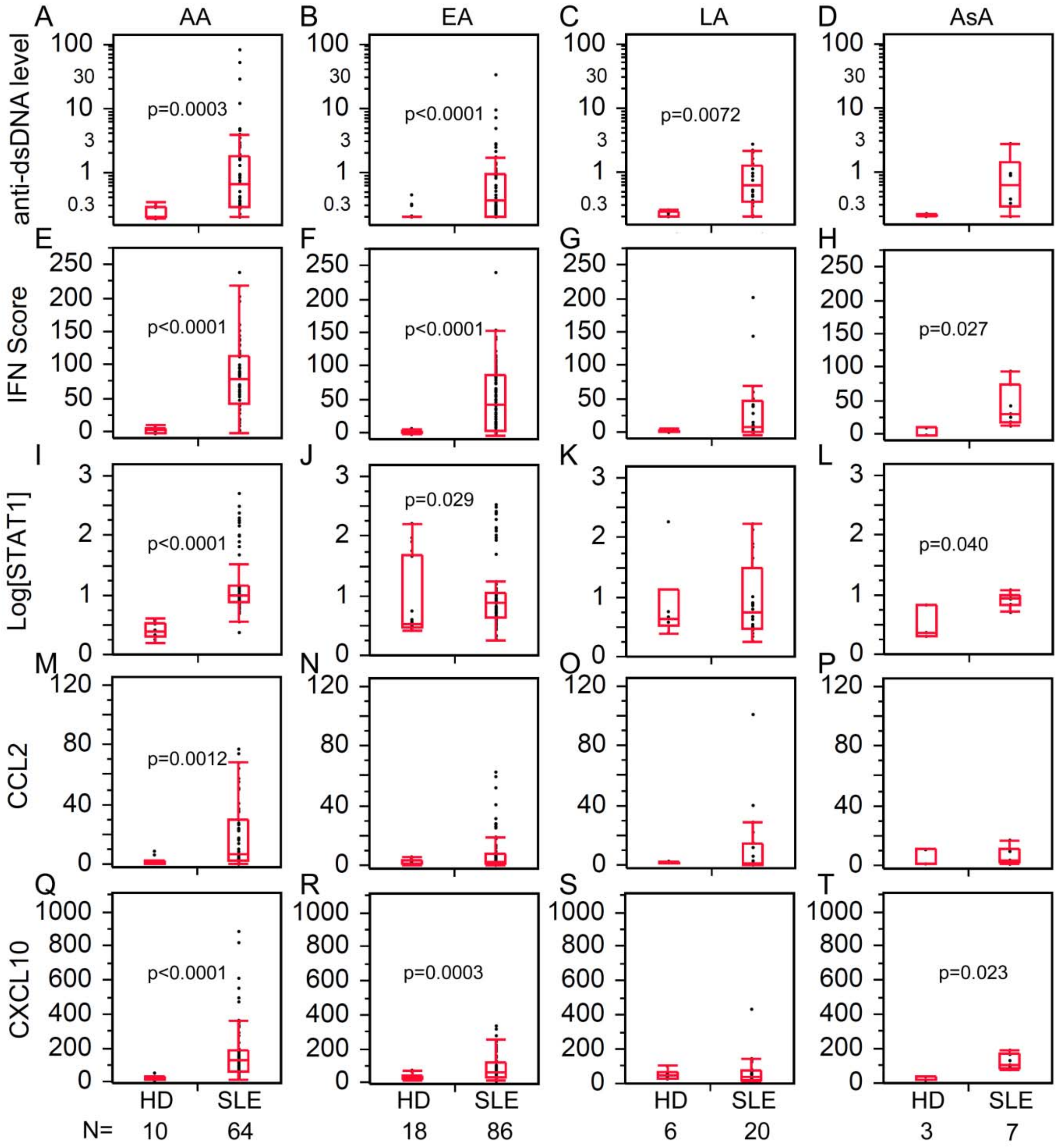

Anti-dsDNA level, IFN score, STAT1, CCL2, and CXCL10 in individuals with different ethnic background. A-D. Anti-dsDNA titer was significantly higher in SLE patient visits of AA, EA, and LA race compared to HD of the respective race. **E-L,Q-T.** IFN score, STAT1, and CXCL10 were higher (*P* ≤0.040) in AA, EA, and AsA patients than HD, while no significant difference was observed for LA . **M-P**. CCL2 was only significantly higher in AA but not in EA, LA, and AsA.

Figure S2

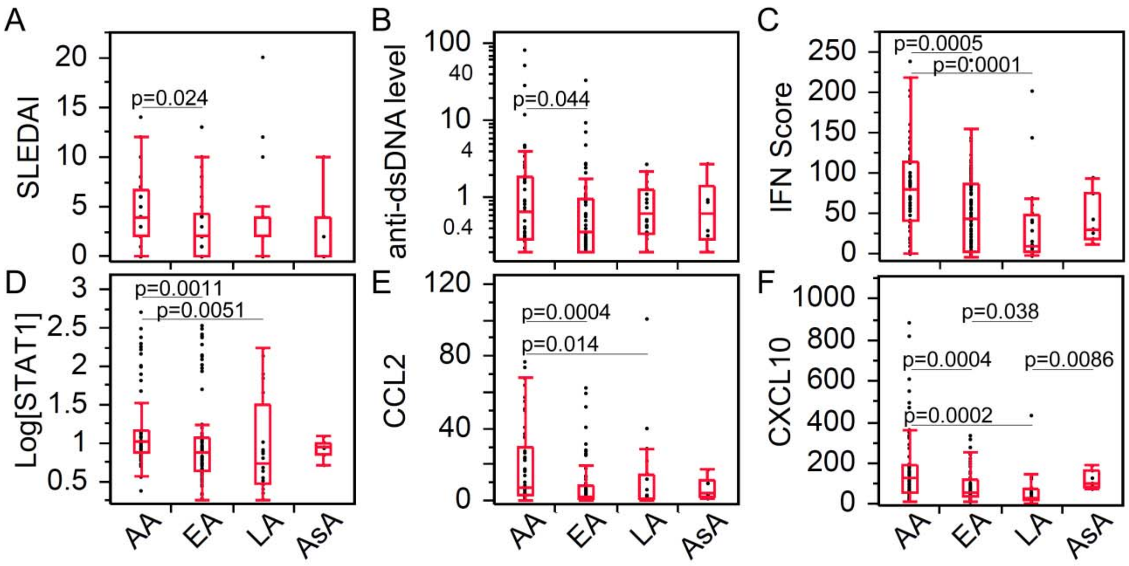

Comparison of SLEDAI, anti-dsDNA titer, IFN score, STAT1, CCL2, and CXCL10 in patients with different ethnic background. To examine whether difference of race could affect clinical and biomarkers, AA, EA, LA, and AsA patient visits were compared to each other. Overall, AA patient visits displayed generally higher SLEDAI (**A**), anti-dsDNA titer (**B**), IFN score (**C**), STAT1 (**D**), CCL2 (**E**), and CXCL10 (**F**) than SLE patient visits of any other races.

Figure S3

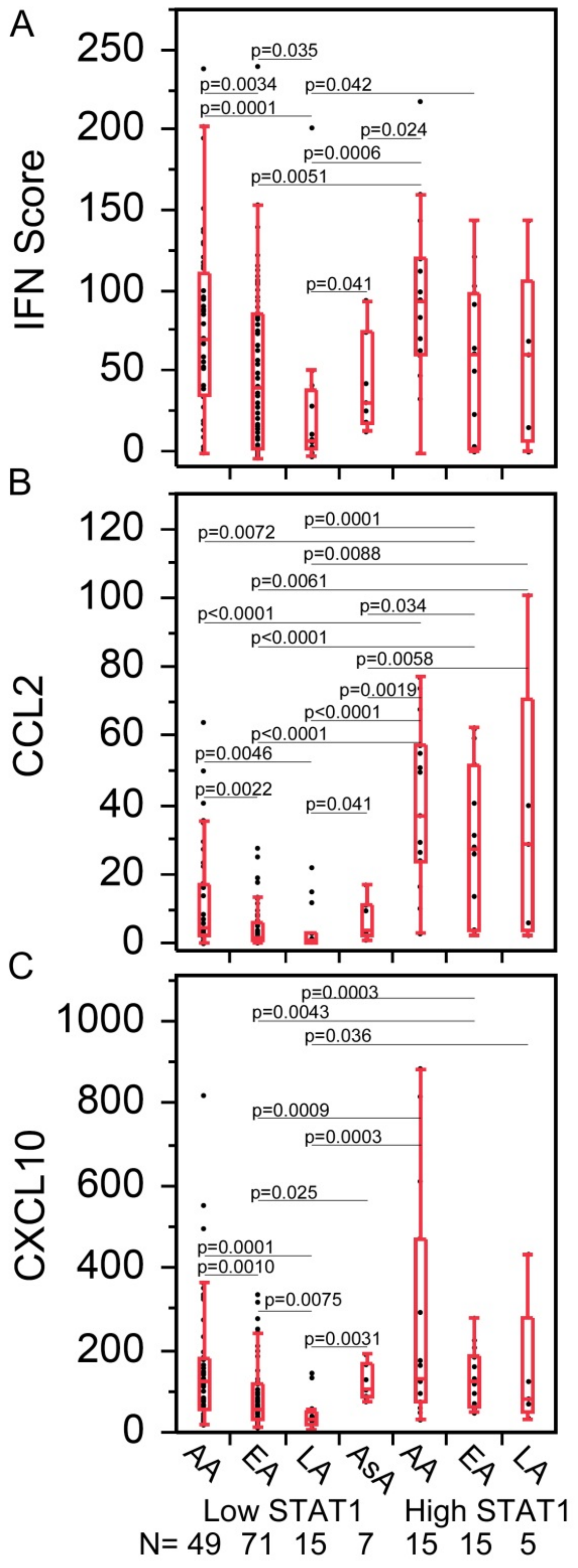

IFN score, CCL2, and CXCL10 in individuals with different ethnic background and STAT1 levels. IFN score (**A**), CCL2 (**B**), and CXCL10 (**C**) levels were compared segregated based on race and high and low STAT1. **A**. IFN scores were not significantly different between high and low STAT1 groups of the same race. In high STAT1 patient visits did not display significant differences among the different ethnic groups. There were too few patients in AsA high STAT1 group to be included. However, in the low STAT1 patient visits, AA were higher than EA and LA, EA were higher than LA, and LA had the lowest levels. **B-C**. High STAT1 patient visits displayed higher CCL2 and CXCL10 compared to their corresponding low STAT1 groups of the same race. Ethnic differences were not observed for AA, EA, and LA in the high STAT1 patients; however, in low STAT1 patient visits, similar difference appeared to have the same effect on CCL2 and CXCL10 expression as described above for IFN scores.

Figure S4

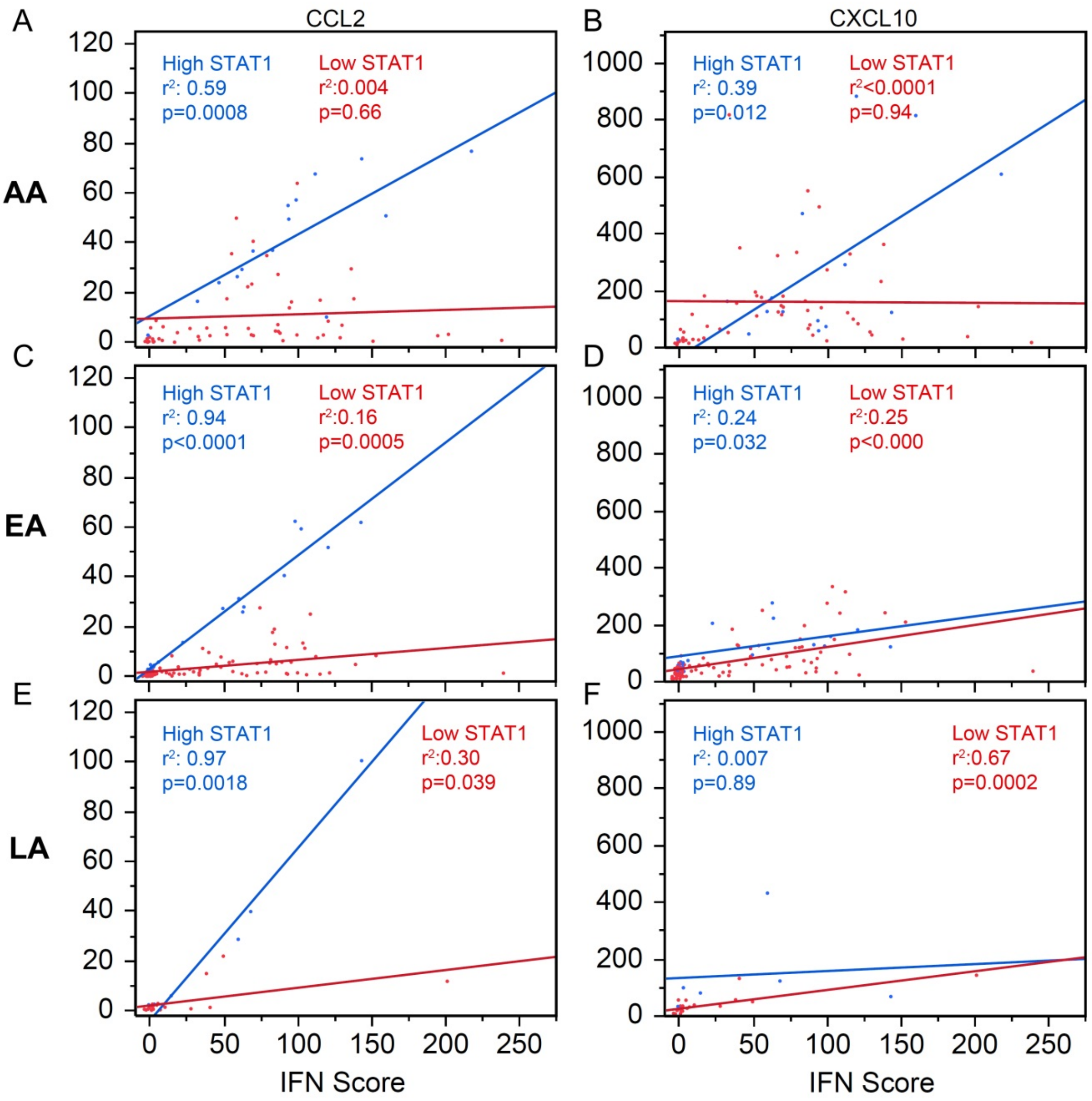

CCL2, CXCL10, and IFN score in individuals with different ethnic background and high vs low STAT1 groups. An analysis similar to that in Figure 7 was performed, but in addition, patient visits were segregated by race and by high and low STAT1. The effects of high and low STAT1 on the association of CCL2 and CXCL10 with IFN score did not appear to be influenced by AA (**A,B**), EA (**C,D**) and LA (**E,F**) ethnicity.
